# Liver single-nucleus multiome profiling reveals cell-type mechanisms for cardiometabolic traits

**DOI:** 10.1016/j.ajhg.2025.11.009

**Published:** 2025-12-02

**Authors:** Abdalla A. Alkhawaja, Kevin W. Currin, Hannah J. Perrin, Swarooparani Vadlamudi, Amy S. Etheridge, K. Alaine Broadaway, Gabrielle H. Cannon, Carlton W. Anderson, Anne H. Moxley, Alina C. Iuga, Erin G. Schuetz, Federico Innocenti, Terrence S. Furey, Karen L. Mohlke

**Affiliations:** 1 Department of Genetics, University of North Carolina, Chapel Hill, NC 27599, USA; 2 Eshelman School of Pharmacy, Division of Pharmacotherapy and Experimental Therapeutics, University of North Carolina, Chapel Hill, NC 27599, USA; 3 Advanced Analytics Core, University of North Carolina, Chapel Hill, NC 27599, USA; 4 Department of Pathology and Laboratory Medicine, University of North Carolina, Chapel Hill, NC, USA; 5 Department of Pharmaceutical Sciences, St. Jude Children’s Research Hospital, Memphis, TN 38105, USA; 6 Department of Biology, University of North Carolina, Chapel Hill, NC 27599, USA

**Keywords:** liver, single nucleus, chromatin accessibility, expression, multiome, quantitative trait loci, caQTL, eQTL, cardiometabolic, lipid, cholesterol, hepatocyte, liver sinusoidal endothelial cell

## Abstract

The liver is a central regulator of cardiometabolic physiology, coordinating processes such as lipid and glucose metabolism, protein synthesis, and detoxification. Genome-wide association studies (GWASs) have identified hundreds of genetic variants associated with cardiometabolic traits, yet their molecular mechanisms in liver cell types remain unclear. Using multiome gene expression and accessible chromatin sequencing on liver samples from 39 individuals, we profiled gene expression and chromatin accessibility in 68,398 nuclei across six primary liver cell types. We identified 306,706 accessible chromatin regions, including 70,884 regions that were undetected in bulk tissue analyses and predominantly represent less abundant cell types. To identify genetic effects on gene regulation in liver cell types, we mapped quantitative trait loci (QTLs) and detected 1,885 chromatin accessibility QTLs (caQTLs) and 67 expression QTLs (eQTLs). We integrated cell-type QTLs with GWAS signals and revealed cell types, genes, and chromatin regulatory elements involved in cardiometabolic traits, such as liver enzyme and cholesterol levels. Non-hepatocyte cell-type QTL analyses exposed previously obscured mechanisms, such as an eQTL for *ADAMTS12* in liver sinusoidal endothelial cells potentially involved in liver fibrosis, demonstrating that single-nucleus approaches can capture regulatory events missed in bulk analyses. Furthermore, we predicted the cell type of action for bulk liver caQTLs colocalized with GWAS signals, enhancing mechanistic insights for complex trait associations. Our findings provide a high-resolution map of the hepatic regulatory landscape and advance the understanding of cellular contexts and molecular mechanisms underlying cardiometabolic traits.

## Teaser

This study used single-nucleus approaches to map genetic variants associated with chromatin accessibility and gene expression across liver cell types. Linking these genetic variants to cardiometabolic trait association signals implicated specific genes and liver cellular contexts in disease risk.

## Introduction

Genome-wide association studies (GWASs) have identified hundreds of signals for cardiometabolic traits,^1^^,^^2^^,^^3^^,^^4^^,^^5^^,^^6^ but the molecular mechanisms and cell type(s) of action for most of these signals have not yet been defined. GWAS signals are enriched in regulatory elements of trait-relevant tissues.^7^^,^^8^^,^^9^ Studies of gene expression quantitative trait loci (eQTLs)^10^ and chromatin accessibility QTLs (caQTLs)^11^^,^^12^^,^^13^^,^^14^ have identified GWAS variants that may regulate gene expression and contribute to phenotypic variation. Profiles of gene expression and chromatin accessibility in single cells or nuclei have been used to predict specific functional variants and identify cell-type regulatory landscapes and target genes at many GWAS signals.^15^^,^^16^^,^^17^^,^^18^^,^^19^ To annotate additional GWAS signals, single-nucleus gene expression and chromatin accessibility profiles are needed in more disease-relevant tissues.

Liver tissue regulates many processes involved in cardiometabolic disease, including lipid and glucose metabolism, inflammation, protein synthesis, and drug detoxification.^20^^,^^21^ Bulk liver tissue eQTLs^22^^,^^23^ and caQTLs^11^^,^^13^ have predicted functional variants, regulatory elements, and target genes at GWAS signals for liver-relevant traits. While differences in gene expression between liver cell types have been reported,^24^^,^^25^^,^^26^^,^^27^ few studies have reported cell-type eQTLs in human liver.^28^^,^^29^ In addition, few studies have analyzed liver cell-type chromatin accessibility.^17^^,^^30^ Cell-type caQTLs have only recently been mapped in human liver.^29^ Single-nucleus gene expression and chromatin accessibility profiles from multiple individuals are needed to identify genetic effects on gene regulation in specific liver cell types.

Here, we jointly profiled chromatin accessibility and gene expression in 68,398 single nuclei from the liver tissue of 39 individuals. We characterized the chromatin accessibility landscape of liver cell types and identified enrichment of GWAS signals in the cell-type chromatin profiles. Leveraging data across multiple individuals, we identified cell-type eQTLs and caQTLs and used them to predict mechanisms of GWAS signals. Finally, we used cell-type accessible chromatin regions to predict the cell types of action for GWAS-colocalized caQTLs from bulk liver tissue.

## Material and methods

### Liver tissue

Human liver tissue was collected from 40 deceased organ donors through the National Institutes of Health Liver Tissue Cell Distribution System (LTCDS). Tissue was obtained from the LTCDS and approved for use in this study as non-human subject research by the institutional review boards (IRBs) at St. Jude Children’s Research Hospital (Memphis, TN) and the University of North Carolina (Chapel Hill, NC). Fibrosis and steatosis were assessed using hematoxylin and eosin (H&E)- and Masson’s trichrome-stained slides from frozen tissue samples fixed in neutral buffered formalin.

### Genotyping and imputation

Genotyping of DNA from the 40 donors in the current study on the Illumina Human610-Quad v.1.0 BeadChip was previously described as part of a larger study of 224 liver donors.^22^ We performed genotype imputation to the TOPMed reference panel v.r2^31^ together with 117 additional liver donors as previously described.^11^ We retained imputed genotypes with an imputation r^2^ > 0.3 using BCFtools v.1.15.1.^32^ We inferred genetic similarity to major 1000 Genomes (1000G) populations using genotype principal components (PCs) as previously described.^11^

### Nuclei isolation

We isolated nuclei from ∼12 mg of frozen human liver tissue for each sample. We pooled aliquots from sets of five individuals, pulverized them using a cell crusher (CellCrusher), lysed cells in ice-cold lysis buffer (10 mM Tris-HCl [pH 7.4], 10 mM NaCl, 3 mM MgCl_2_, 1% BSA, 0.1% Tween 20, 0.1% Nonidet P40, 0.01% digitonin, 1 mM dithiothreitol, and 0.04 U/μL RNase inhibitor) with vigorous shaking for 5 min at 4°C, and homogenized them using a 2-mL dounce with 10 strokes of the loose pestle followed by 30 strokes of the tight pestle. We centrifuged the homogenized samples at 500 × *g* for 5 min at 4°C, resuspended them in 1 mL ice-cold wash buffer (10 mM Tris-HCl [pH 7.4], 10 mM NaCl, 3 mM MgCl_2_, 1% BSA, 0.1% Tween 20, 1 mM dithiothreitol, and 0.04 U/μL RNase inhibitor), sequentially filtered them through 70-, 30-, and 20-μm filters, centrifuged them at 500 × *g* for 5 min at 4°C, and resuspended them in 300 μL wash buffer. We then washed nuclei suspensions twice by layering them over 300 μL of 1.5 M sucrose and centrifuging them at 1,000 × *g* for 10 min at 4°C. We resuspended final nuclei pellets in 1 mL wash buffer, centrifuged them at 500 × *g* for 5 min at 4°C, and resuspended them in 35 μL of diluted nuclei buffer (1× 10× Genomics Nuclei Buffer, 1 mM dithiothreitol, and 1 U/μL RNase inhibitor).

### 10× multiome assays

We performed single-nucleus chromatin accessibility and gene expression profiling using the 10× Genomics Chromium Controller and Chromium Next GEM Single Cell Multiome ATAC + Gene Expression Reagent Bundle following the manufacturer’s instructions. Briefly, we stained nuclei with acridine orange and propidium iodide (Logos Biosystems PN-F23001) and assessed them for viability, concentration, and singleness using a LUNA-FL Dual Fluorescence Cell Counter (Logos Biosystems). We loaded 20,000 nuclei per inlet on a Chromium Controller instrument (10× Genomics). We purified sequencing libraries using SPRIselect beads, visualized them using the Bioanalyzer High Sensitivity DNA chip, and quantified them via the KAPA Library Quantification Kit for Illumina Platforms (Roche PN-KK4824). We separately pooled assay for transposase-accessible chromatin sequencing (ATAC-seq) and RNA sequencing (RNA-seq) libraries, spiked them with 1% PhiX sequencing control (Illumina), and sequenced them on a NovaSeq 6000 S4 array at the UNC High Throughput Sequencing Facility in paired-end format to a total depth of ∼8 billion read pairs passing quality filters. We used CellRanger-ARC Count v.2 (10× Genomics) to align sequencing reads to the GRCh38-2020-A human reference genome, using the filtered GENCODE v.32^33^ GTF file for gene quantification.

### Identification of singlet droplets and donor deconvolution

To prepare genotype VCF files, we selected single-nucleotide polymorphisms (SNPs) that overlapped gene coordinates from the Cell Ranger GTF file using BCFtools v.1.15.1^32^ and BEDTools v.2.29.^34^ To prepare BAM files, we selected unique alignments (mapq = 255 from STAR) using samtools v.1.15.1,^32^ selected alignments overlapping SNPs using BEDTools v.2.29,^34^ and reordered chromosomes to match the order in the VCF file using the ReorderSam tool from Picard (http://broadinstitute.github.io/picard). We performed genotype-based donor deconvolution and singlet identification using demuxlet^35^ as implemented in popscle (https://github.com/statgen/popscle). We ran demuxlet separately per multiome well using barcodes with at least 1,000 RNA unique molecular identifiers (UMIs) and using the parameters –field GT –min-BQ 20 –min-MQ 30 –min-mac 4 –min-callrate 0.95. To test for sample swaps between wells, we provided the full list of 40 donors to the –sm-list option for each demuxlet run. We retained barcodes that demuxlet classified as singlets and assigned them to the best-matched donor.

### Nuclei filtering and cell-type annotation

We applied several quality control (QC) filters to select high-quality nuclei from the set of singlet barcodes. For RNA, we selected barcodes with ≥1,000 UMIs, ≥500 expressed genes, and ≤10% of reads mapping to the mitochondrial chromosome using Seurat v.5.^36^ For ATAC, we counted reads into a set of bulk liver tissue ATAC peaks mapped in 138 individuals^11^ and selected barcodes with >500 fragments in peaks, >15% fragments overlapping peaks, and transcription start site (TSS) enrichment >2 (calculated from EnsDb.Hsapiens.v86) using Signac.^37^

We used DecontX^38^ to minimize ambient RNA contamination. To generate a set of cell-type labels, we clustered nuclei by their gene expression profiles separately per batch (set of nuclei pooled from 5 individuals) using Seurat.^36^ We log-normalized gene counts, calculated PCs using the 2,000 most variable genes, constructed shared nearest-neighbor graphs using the first 20 PCs, identified clusters using the Louvain algorithm (resolution = 0.25), and generated uniform manifold approximation and projection (UMAP) embeddings using the 20 PCs. We assigned clusters to cell types based on established cell-type markers.^27^ We observed multiple hepatocyte clusters, which we collapsed to a single cell type. We then ran DecontX separately by batch using these cell types and defining barcodes with ≤100 UMIs as the background. Next, we re-applied the gene expression filters, which resulted in 39 samples with nuclei that passed QC.

Following decontamination, we merged corrected count matrices from all batches and identified clusters. To account for batch effects, we calculated 50 PCs from the decontaminated RNA data and used Harmony^39^ as implemented in the Seurat IntegrateLayers function. Next, we recalculated clusters and UMAP projections and annotated the clusters. To obtain more granular cell-type annotation, we used Azimuth (https://azimuth.hubmapconsortium.org/) in R with the supplied liver dataset as the reference to transfer annotations to our gene expression data.

### Cell-type accessible chromatin regions

We used the barcodes of the gene-expression-based annotated nuclei to subset the Cell Ranger ATAC-seq BAM files by cell type and donor combination and generate pseudobulk BAM files. We selected non-duplicate reads with high mapping quality to primary nuclear chromosomes using samtools v.1.17^32^ with the parameters -f 3 -F 4 -F 8 -F 256 -F 2048 -F 1024 -q 30. Due to the low read depth, we merged BAM files across donors within a cell type using samtools^32^ prior to peak calling. We then converted the merged BAM files to BED using BEDTools v.2.29^34^ and called peaks for each cell type using MACS2^40^ v.2016-02-15 with the parameters –nomodel –shift −100 –extsize 200 -q 0.05 –keep-dup all; the –keep-dup all option was used because duplicate removal at the nucleus level was previously performed using samtools, and we wanted to retain reads mapping to the same genomic location but in different nuclei. We removed peaks overlapping ENCODE exclusion regions^41^ using BEDTools.^34^ We used ataqv v.1.0.0^42^ to calculate the quality metrics of the pseudobulk datasets, including TSS enrichment and the percentage of reads overlapping peaks. We generated a single peak set by merging peaks across cell types using BEDTools.^34^

To cluster nuclei using their chromatin accessibility profiles, we normalized counts using the TF-IDF function and used all peaks to calculate 50 latent semantic indexing (LSI) components. We computed shared nearest-neighbor graphs, clusters, and UMAP projections as described for the gene expression analysis, excluding the first LSI component, as it correlated with library size. To account for batch effects, we ran Harmony^39^ on 50 LSI components. To integrate the gene expression and chromatin accessibility UMAP projections, we computed weighted nearest neighbors (WNNs) using 50 gene expression PCs and 49 chromatin accessibility LSI components (excluding the first). We used the gene expression annotations for cell-type identification.

To identify cell-type peaks that were missed in analyses of bulk tissue, we determined which cell-type peaks shared at least 1 base pair (bp) with previously reported bulk liver tissue ATAC-seq peaks^11^ using the plyranges R package.^43^

### Cell-type marker ATAC-seq peaks

We used differential chromatin accessibility analysis to identify marker peaks for each cell type. To compare hepatocyte nuclei to other cell types, we downsampled the number of hepatocyte nuclei in each donor to 10% using the sample function in R^44^ (random number generator seed of 123). We then called peaks on the downsampled hepatocytes and merged peaks across cell types using downsampled hepatocytes instead of full-depth hepatocytes. We counted the number of ATAC-seq reads from the pseudobulk BAM files that overlapped the merged cell-type peaks using featureCounts,^45^ corrected for peak-level GC content using Exploratory Data Analysis and Normalization for RNA-seq (EDASeq),^46^ and corrected for library size using DESeq2^47^ size factors. We only tested peaks with a minimum mean count of 10 across donors in a cell type for marker peak status in that cell type. We tested for differentially accessible peaks between each pair of cell types using DESeq2^47^ and defined marker peaks as peaks more accessible (false discovery rate [FDR] < 5%, Benjamini-Hochberg [BH] procedure^48^) in one cell type compared to all other cell types.

We used the Genomic Regions Enrichment of Annotations Tool (GREAT)^49^ to test if marker peaks were located near genes involved in cell-type-relevant biological functions using the GO Biological Process ontology^50^ and using all merged cell-type peaks as the background. We tested if marker peaks were enriched for known transcription factor (TF) motifs using HOMER v.4.11^51^ with the -size 200 parameter and using all merged cell-type peaks as the background.

### Heritability enrichment in cell-type peaks

We downloaded GWAS summary statistics from the Benjamin Neale lab analysis of the UK Biobank dataset (https://nealelab.github.io/UKBB_ldsc/downloads.html). We tested for heritability enrichment for traits with heritability *Z* scores ≥4 in cell-type ATAC peaks using stratified linkage disequilibrium (LD) score regression implemented in the LDSC software.^9^^,^^52^ We ran a single LDSC model that consisted of 7 annotations: annotations for peaks in each of our identified 6 cell types (excluding B cells) and the baseline v.1.2 annotation provided with LDSC. To run LDSC, we used liftOver^53^ to convert cell-type peak coordinates to GRCh37, used the GRCh37-based LDSC reference files derived from 1000G phase 3 European genotypes, and performed analysis using HapMap SNPs. For each combination of trait and cell-type peak annotation, we calculated the *p* value of the LDSC coefficient *Z* score using a one-sided test assuming a standard normal distribution. We calculated the FDR of the coefficient *p* values using the BH procedure^48^ and classified the results with an FDR < 5% as significant.

### Mapping caQTL

We removed ATAC-seq reads exhibiting allelic mapping bias from the pseudobulk BAM files per cell type and donor using the remapping pipeline in WASP v.0.3.4^54^ with updates as of February 9, 2023. Because the Cell Ranger alignment pipeline cannot be run in the WASP pipeline, we approximated the Cell Ranger alignment command using BWA-mem v.0.7.17^55^ with the parameters -M -I 250,150. For each cell type, we generated a peak-by-donor count matrix using featureCounts,^45^ corrected for peak-level GC content using EDASeq,^46^ and corrected for library size and performed variance stabilization using DESeq2.^47^ We performed principal-component analysis (PCA) on variance-stabilized peak counts and found that PC values were strongly driven by the fraction of 0 counts in each sample. Therefore, we did not use ATAC-seq PCs as covariates in the caQTL model. Instead, we used the first two genotype PCs, sex, and ATAC TSS enrichment as caQTL covariates. We only tested peaks for caQTLs if the peaks had at least 5 counts in at least 10 donors for a given cell type. We used FastQTL^56^ to test for associations between inverse normal-transformed peak counts and bi-allelic variants within 1 kilobase (kb) of peak centers and with a minor-allele frequency (MAF) of at least 0.1 in the 39 donors that passed QC. We corrected the FastQTL beta-adjusted *p* values for genome-wide multiple testing using the BH procedure and defined peaks with a significant caQTL (FDR < 5%) as caPeaks.

We classified a cell-type caPeak as shared with a caPeak in bulk liver tissue^11^ if the peaks overlapped by at least 1 bp and if their lead variants were in LD (r^2^ ≥ 0.5, TOPMed^31^ Europeans calculated by TOPLD^57^). We used r^2^ ≥ 0.5 to define “shared” signals across the study, selecting this threshold as a practical screen given the imprecise identification of leads in small sample sizes. We also report shared signals at r^2^ ≥ 0.8. We compared effect sizes of the shared cell-type and bulk caQTL signals with the same lead variants and assessed their correlation using Pearson’s R^2^.

### Cell-type caQTLs shared with bulk GTEx eQTLs and repressor motifs

We considered a cell-type caQTL to be shared with a bulk liver tissue eQTL from GTEx v.8^10^ if their lead variants were in strong LD (r^2^ ≥ 0.5, TOPMed Europeans^31^^,^^57^). We compared caQTL-eQTL signals that only exhibited an opposite effect direction to signals that only exhibited the same effect direction. We retained caPeaks if the lead variant or a proxy variant (LD r^2^ ≥ 0.5) overlapped the peak, and we used liftOver^53^ and BEDTools^34^ to compare variants to locations from the ENCODE Motif Browser^58^ of 103 motifs for repressor TFs in HepG2.^59^

### Mapping eQTL and power analysis

To prepare the data for eQTL analysis, we generated pseudobulk expression profiles by summing the count matrices across cells for each cell type-donor combination using the AggregateExpression function from Seurat v.5.^36^ Per cell type, we kept genes with at least 10 counts in at least 10 donors. We corrected for library size and performed variance stabilization using DESeq2.^47^ We used FastQTL^56^ to test for association of inverse normal-transformed gene counts and bi-allelic variants within 1 megabase (Mb) of the TSS of a gene and with a MAF ≥ 0.1. We included sex, two genotype PCs, and a variable number of expression PCs as covariates. Specifically, we included the first 2, 3, 6, 2, 3, and 6 PCs for hepatocytes, liver sinusoidal endothelial cells (LSECs), Kupffer cells, mesenchymal cells, natural killer (NK)-T cells, and cholangiocytes, respectively, which maximized the number of genes with significant eQTLs.

We adjusted the FastQTL beta-adjusted *p* values for genome-wide multiple testing using the BH procedure and classified genes with a significant eQTL (FDR < 5%) as an eGene. When multiple eQTLs were identified for the same eGene across cell types, we clumped eQTLs based on LD (r^2^ ≥ 0.2), which grouped 79 unclumped eQTLs into 67 distinct eQTLs.

We considered a cell-type eQTL to be shared with a bulk liver tissue eQTL from GTEx v.8^10^ if the eGenes shared the same Ensembl ID or gene symbol and the lead variants were in strong LD (r^2^ ≥ 0.5, TOPMed Europeans^31^^,^^57^). For cases in which the GTEx lead variant was not present in the TOPMed panel, we calculated the LD between our cell-type lead variant and the GTEx second-best variant ranked by *p* value. We assessed the correlations of these shared signals by comparing the effect sizes of the cell-type eQTL lead variants in both the single nucleus and GTEx using Pearson’s R^2^.

To estimate the effect of sample size and number of nuclei per individual on eQTL detection power, we used scPower,^60^ which incorporates expression probability with eQTL power. We used our cell-type gene expression matrices to estimate gene expression prior probabilities. As no large-scale liver single-cell eQTL studies exist, we used effect sizes from bulk liver GTEx eQTLs as priors. We estimated eQTL power for high- (hepatocytes), mid- (LSEC), and low- (cholangiocytes) abundance cell types using the power.sameReadDepth.restrictedDoublets function. Sample sizes were varied from 39 (current study) to 400, with a number of nuclei per individual (nCells) of 2,000–8,000. Cell-type frequencies (ct.freq) matched those of each cell type, and the multiplet rate was fixed at 0.56. Expressed genes were defined as min.UMI.counts = 10 and perc.indiv.expr = 10 (i.e., 10 individuals). The power for genes with expression ≤100 was non-parametrically simulated. Results are presented as relative fold changes to our study of 39 samples and 4,000 nuclei/individual because the model’s absolute predictions did not match our empirical data.

### Identifying cell-type QTLs shared with GWAS signals

We retrieved published GWAS summary statistics and signal lead variants for 17 cardiometabolic traits, including lipids,^1^ coronary artery disease,^2^ plasma liver enzyme levels,^3^ glycemic traits,^4^ type 2 diabetes,^5^ waist-hip ratio adjusted for body mass index (BMI),^6^ BMI,^61^ vitamin D,^62^ and C-reactive protein.^63^ We used summary statistics from individuals genetically similar to the European population for all GWASs. When studies did not provide conditionally distinct signals, we used those previously identified in our prior analyses.^23^ To more comprehensively capture the LD structure of the secondary signal at *XKR9*, which lies at the edge of the locus boundary, we extended the signal window to 1 Mb upstream of the second signal lead variant before running the conditional analysis.

### Cell-type QTLs shared with signals in the GWAS Catalog

We retrieved signal lead variants from the NHGRI-EBI GWAS Catalog^64^ (accessed June 4, 2025). For each trait, we retained variants with available rsIDs or genomic coordinates and selected those with the lowest *p* value per trait. Cell-type eQTLs and caQTLs were considered to be shared with GWAS signals when LD r^2^ ≥ 0.5.

### Annotating bulk caQTLs with cell-type peaks

We retrieved published bulk liver caPeaks and their predicted target genes and GWAS colocalizations.^11^ The predicted target genes were linked to bulk liver caPeaks using four approaches: TSS proximity, caQTL-eQTL colocalization, promoter high-throughput chromatin confirmation capture (Hi-C), and shared caQTL signals for distal and promoter bulk peaks. We determined which cell-type peaks overlapped by at least 1 bp with bulk liver caPeaks using BEDTools intersect.^34^ When plotting the example at the *GAS6* locus, we observed that the bulk liver ATAC peak (bulk peak94548) had two distinct bulk caQTL signals; thus, we performed caQTL mapping for this peak, including genotype dosage of one of them, rs74118417, as an additional covariate to the covariates used in the bulk study^11^ and used this conditioned signal in plotting.

## Results

### The cell-type regulatory landscapes of the human liver

**Figure 1:**
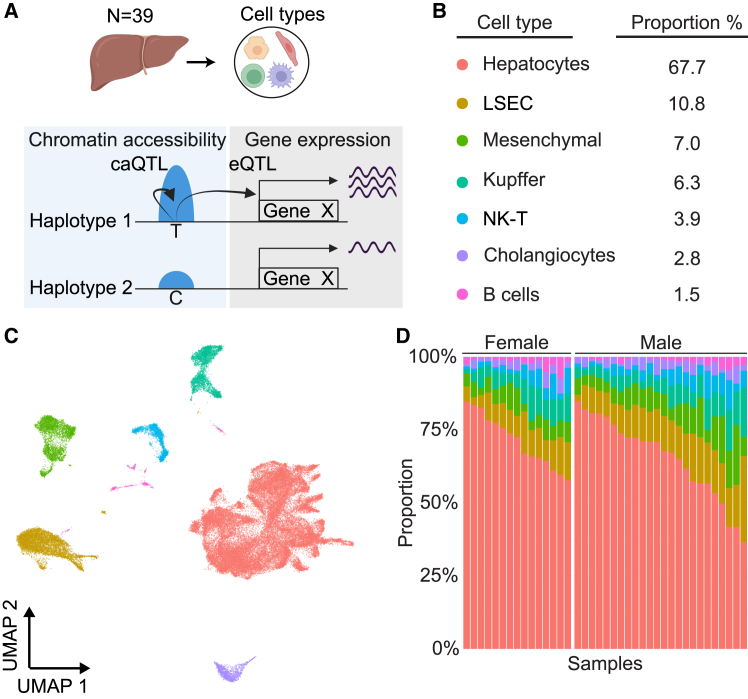
Identification and characterization of major cell types in human liver tissue using single-nucleus RNA and ATAC sequencing (A) Project overview. Nuclei extracted from 39 human livers were sequenced to profile the chromatin and gene expression landscapes, identify eQTLs and caQTLs, and determine which signals are shared with GWAS. Created in BioRender. (B) Cell-type proportions of seven cell populations identified from snRNA-seq analysis of 68,393 nuclei. LSEC, liver sinusoidal endothelial cells; NK-T, natural killer T cells. (C) Joint UMAP of integrated snRNA-seq and snATAC-seq profiles showing seven main cell populations. The colors correspond to the cell types shown in (B). (D) The cell-type composition of each sample, separated by sex. The colors correspond to the cell types shown in (B).

To profile the regulatory elements and gene expression in cell types of the human liver, we performed single-nucleus ATAC-seq (snATAC-seq) and RNA-seq (snRNA-seq) on 40 genotyped liver samples (Figure 1; Table S1). After rigorous QC, we identified 68,398 nuclei from 39 samples, averaging 1,753 nuclei per sample (Figures S1 and S2). Using established cell-type gene expression markers^27^ (Figure S3), we identified seven broad cell types: hepatocytes, LSECs, mesenchymal cells, Kupffer cells, NK-T cells, cholangiocytes, and B cells (Figure 1). Prior to batch correction, hepatocytes showed sample-specific clustering (Figures S4–S6), consistent with earlier findings.^24^^,^^27^ Although reference liver single-cell RNA-seq data^24^^,^^25^^,^^26^^,^^65^^,^^66^ enabled finer subtype classifications for non-parenchymal cells (Figure S7), we maintained broad cell-type definitions for downstream QTL analyses to maximize detection power. Cell-type proportions varied considerably between samples (Figure 1). Hepatocytes comprised the majority of nuclei at 68% (range: 36%–85%; Table S1), while B cells were extremely sparse (1.5% of nuclei; Figure 1). Due to their low abundance, we excluded B cells from further analyses. These well-defined cell-type profiles lay the groundwork for understanding the cellular context of gene regulation in the liver.

Because each nucleus was profiled using snRNA-seq and snATAC-seq, the gene-expression-based cell-type annotations apply to both modalities. To map the cell-type regulatory landscapes of the human liver, we identified 306,706 accessible chromatin regions (peaks) across the six cell types (Data S2). Cell clustering based on peak accessibility recapitulated gene-expression-based cell-type clusters, indicating concordance between the two profiling methods (Figure S5). We identified 17,147 cell-type marker peaks that were more accessible in one cell type compared to all others (FDR < 0.05; Figure S8; Table S2; Data S3). These marker peaks were enriched near genes involved in canonical cell-type processes: lipid, carbohydrate, and drug metabolism genes for hepatocytes; angiogenesis and blood vessel morphogenesis genes for LSECs; and extracellular matrix organization genes for mesenchymal cells (Figure S8; Table S3). Consistent with these functional associations, marker peaks were enriched for binding motifs of known cell-type TFs, including HNF4A and the HNF1 family for hepatocytes, SPI1 for Kupffer cells, and SP1 for cholangiocytes (Figure S8; Table S4). Together, these results demonstrate that we generated a high-quality cell-type-resolved map of accessible chromatin profiles in human liver tissue.

**Figure 2:**
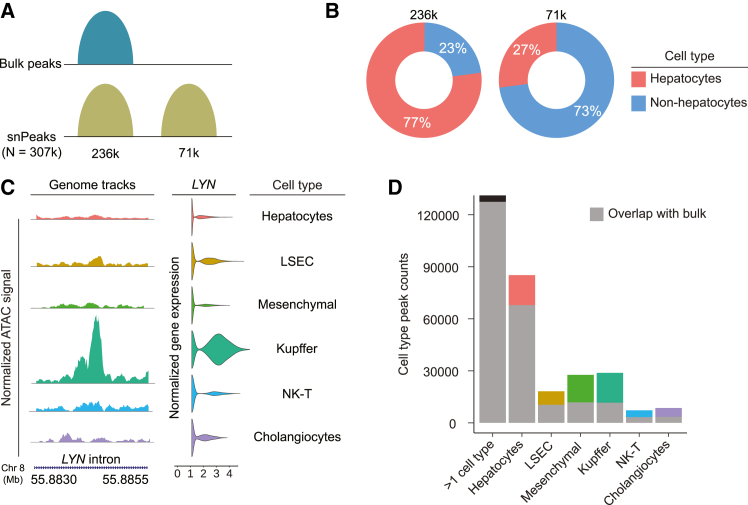
Accessible chromatin in liver cell types (A) Among 306,706 accessible chromatin peaks observed in liver cell types using snATAC-seq, 235,822 overlapped accessible chromatin peaks detected in bulk liver ATAC-seq. (B) Among cell-type peaks that were detected in bulk liver ATAC-seq, only 23% were not detected in hepatocytes. Among peaks not detected in bulk liver ATAC-seq, 73% were not detected in hepatocytes. (C) An example of a cell-type marker peak in an intron of *LYN*. Coverage plot showing normalized ATAC-seq signal across cell types, with a Kupffer cell peak that did not pass thresholds of detection in other cell types or bulk liver tissue. The adjacent violin plots represent *LYN* gene expression by cell type. (D) Distribution of peaks detected in each cell type. Portions of the bars shaded in gray represent peaks that were also detected in bulk liver. Portions of the bars shaded in colors represent peaks only detected using snATAC-seq.

To investigate the differences in regulatory element discovery between snATAC-seq and bulk ATAC-seq, we compared cell-type peaks to 349,685 bulk liver ATAC peaks from a larger cohort of 138 deeply sequenced individuals.^11^ We found 235,822 cell-type peaks overlapped with bulk, while 70,884 peaks did not (Figure 2). As hepatocytes are the predominant cell type in the liver, we expected that most bulk peaks would originate from that cell type, and indeed, 77% of cell-type peaks that were found in the bulk data were also detected in hepatocytes. In contrast, of the 70,884 cell-type peaks not found in the bulk analysis, only 27% were detected in hepatocytes (Figure 2; Data S2), showing that cell-type ATAC-seq can identify peaks in less abundant cell types that are poorly represented in bulk ATAC-seq data. For example, a cell-type marker peak located in an intron of the Kupffer cell marker gene *LYN* was not detected in the bulk data nor in hepatocytes (Figure 2). *LYN* encodes a member of the Src family of protein tyrosine kinases and plays a role in macrophage inflammatory response.^67^ The 51,717 non-hepatocyte peaks not identified in the bulk analysis demonstrate the added resolution of snATAC-seq to uncover regulatory regions in less abundant liver cell types, enabling a more complete annotation of the hepatic regulatory landscape.

### Cell-type chromatin accessibility and gene eQTLs

To investigate regulatory genetic variation across liver cell types, we conducted QTL analyses for both chromatin accessibility and gene expression. We identified variants significantly associated with chromatin accessibility for 1,885 peaks (caPeaks; FDR < 0.05), nearly entirely in hepatocytes (Tables 1 and S5). To confirm the robustness of these cell-type caQTLs, we compared cell-type caPeaks with caPeaks detected in bulk liver tissue^11^: 90% overlapped with bulk caPeaks. Cell-type and bulk caQTLs showed highly correlated effect sizes (Pearson’s R^2^ = 0.94; Figure S9). Of the 188 cell-type caPeaks detected only in our dataset, 54 were not detected as bulk peaks (Table S6). The identification of these novel regulatory elements highlights the power of snATAC-seq to resolve functional regions missed by bulk profiling. In parallel, we identified 67 eQTLs (FDR < 0.05) across the six liver cell types (Tables 1 and S7). We found 47 cell-type eGenes in common with bulk liver eQTLs in GTEx,^10^ 38 of which (80%) shared the same eQTL signal (lead variants r^2^ ≥ 0.5, 35 at r^2^ ≥ 0.8) (Figure S9; Table S8). The effect sizes of these shared signals were highly correlated between the single-nucleus and bulk liver studies (Pearson’s R2 = 0.94; Figure S9). A notable example includes the well-validated eQTL for *SORT1* detected in hepatocytes.^68^ Together, these results underscore the reproducibility of the cell-type QTL data.

**Table 1:** Chromatin accessibility QTLs and expression QTLs identified in each cell type

**Cell type**	**caQTL count**	**eQTL count**
Hepatocytes	1,880	55
Endothelial	4	8
Kupffer	1	7
Mesenchymal	0	4
Cholangiocytes	0	3
NK-T	0	2
Total	1885	79

Some eQTLs are called in >1 cell type. Clumping by LD r^2^ > 0.2 results in 67 eQTLs.

**Figure 3:**
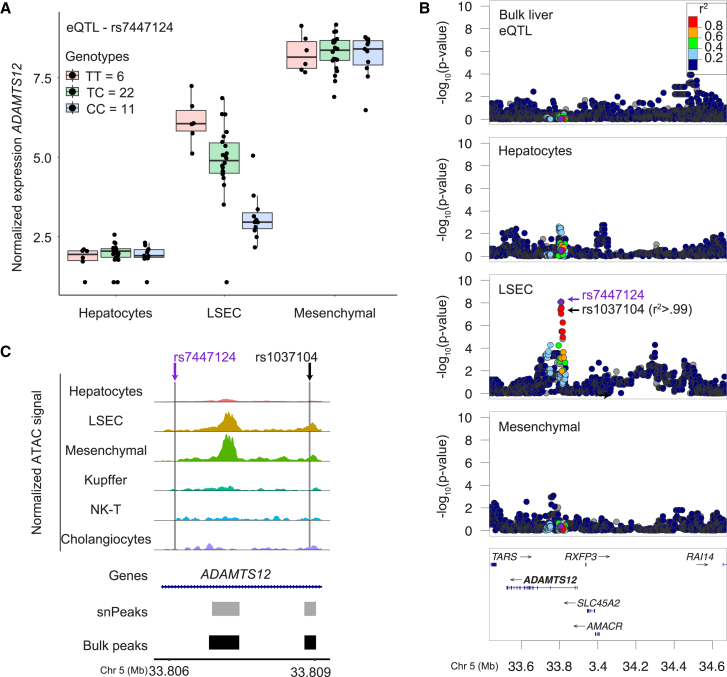
eQTLs for *ADAMTS12* detected in LSECs (A) rs7447124 is associated with *ADAMTS12* expression in LSECs but not in hepatocytes or mesenchymal cells. Boxplots show normalized expression levels by genotypes, with individual points representing samples. (B) Variant association with *ADAMTS12* in bulk liver from liver GTEx v.8 and by cell type. Variants are colored by linkage disequilibrium with lead variant rs7447124 (purple dot). rs1037104 (black arrow) is in high LD with the lead variant (r^2^ > 0.99). (C) rs1037104 is located in an intronic *ADAMTS12* accessible chromatin peak that only passed the detection threshold in LSECs. The gray bars on the bottom indicate regions defined as peaks in one or more cell types, and black bars indicate regions defined as peaks in bulk liver tissue.

Cell-type analysis offers the potential to identify eQTL signals in less abundant cell types. We investigated whether there were cell-type eQTLs not identified in hepatocyte or bulk data and found 12 eQTLs in non-hepatocyte cell types. For example, LSECs represent 11% of liver cells (Figure 1), where they maintain inactivation of hepatic stellate cells (a mesenchymal cell subtype), exerting anti-fibrotic effects.^69^ LSECs could also directly contribute to liver fibrosis by producing fibronectin and modulating extracellular matrix metabolism.^70^ We found an LSEC eQTL for *ADAMTS12*, a gene whose absence is associated with increased fibrosis in mice.^71^ *ADAMTS12* is not identified as an eQTL in hepatocyte or bulk tissue analyses (Figure 3; Table S8), potentially due to the low proportion of LSECs in bulk liver tissue. This LSEC eQTL has a proxy variant (rs1037104, r^2^ = 0.99) located within an intronic accessible peak detected only in LSECs. This signal provides evidence of a genetic regulatory mechanism for *ADAMTS12* in LSECs. Thus, cell-type eQTLs can detect signals and elucidate regulatory mechanisms missed in less abundant cell types and bulk analyses.

To link genetically regulated peaks to genetically regulated genes, we identified cell-type caQTL signals shared with bulk liver eQTLs. We identified caQTLs for 323 hepatocyte caPeaks that were shared (r^2^ ≥ 0.5) with bulk liver eQTLs for 344 genes (240 caPeaks for 243 genes at r^2^ ≥ 0.8); some regulatory elements may influence the expression of more than one gene. 80% of the shared cell-type caQTL-bulk liver eQTL signals showed alleles associated with both increased chromatin accessibility and increased gene expression, suggesting that these regulatory elements primarily function as promoters or enhancers. Conversely, the 20% of shared signals exhibiting opposite effects may represent transcriptional silencers or repressive elements in the hepatocyte regulatory landscape, a pattern that aligns with previous studies.^11^^,^^13^^,^^72^ For caQTL signals that showed the opposite versus shared direction of effect with eQTLs, we compared caQTL variants to the locations of motifs for predicted repressor TFs in HepG2 hepatocytes.^59^ Among 38 caQTL-eQTL signals that showed opposite directions of effect, 2 (5.3%) had variants overlapping repressor motifs, and among 210 caQTL-eQTL signals that showed the same directions of effect, 12 (5.7%) had variants overlapping repressor motifs (Table S10). The lack of over-representation of repressor motifs may be due to the small number of signals tested, the under-annotation of repressor motifs, and/or the presence of some false positive colocalizations. Taken together, cell-type caQTLs predict regulatory elements and their cell type of action for bulk eQTLs.

As the majority of cell-type QTLs were detected in the most abundant cell type, hepatocytes, the absence of significant QTLs in the less abundant cell types likely reflects insufficient detection sensitivity due to lower cell numbers rather than a true biological absence of regulatory effects. While our data reveal numerous genetic effects in hepatocytes, these findings do not establish definitive cell-type specificity. To understand how sample size and the number of nuclei affect the power to detect eQTLs, we ran a power analysis and found that increasing the sample size was generally more effective for increasing eQTL power than increasing the number of nuclei per sample. For example, doubling from the current study sample size was projected to increase the number of detected eQTLs by ∼280% among hepatocytes, LSECs, and cholangiocytes. However, increasing the cell numbers provided a benefit only for less abundant cell types; doubling the number of nuclei per person was estimated to increase eQTL discovery by 130% in cholangiocytes, 10% in LSECs, and none in hepatocytes (Figure S10).

### Predicting cell-type context for disease associations

To determine if genetic risk for certain disease-relevant traits is concentrated in specific liver cell types, we tested for heritability enrichment of GWAS traits in cell-type accessible chromatin peaks using stratified LD score regression. We found that loci associated with plasma levels of liver enzymes were enriched (FDR < 0.05) in hepatocytes, LSECs, cholangiocytes, and Kupffer cells (Figure S11; Table S11). Hepatocytes were enriched in loci for diagnosed diabetes and proteins produced by the liver, such as insulin-like growth factor 1. Kupffer and NK-T cells were enriched in loci for counts of leukocytes and other immune cell types, consistent with their role in immunity. Mesenchymal cells were enriched in loci for blood pressure and eye measurement traits, potentially related to the role of hepatic stellate cells in vitamin A homeostasis.^73^ These results suggest that accessible chromatin regions in different cell types are important for different traits.

To connect regulatory elements to GWAS signals, we identified shared signals between cell-type caQTLs and GWAS signals for 17 cardiometabolic and liver-relevant traits. Among 1,880 hepatocyte caQTLs, 205 were shared with at least 1 GWAS signal (r^2^ ≥ 0.5, 125 caQTLs at r^2^ ≥ 0.8) (Tables 2 and S9). For example, 13% of GWAS signals for plasma levels of gamma-glutamyl transferase (GGT), an enzyme whose elevated levels are a marker of liver damage, and 8% of GWAS signals for low-density lipoprotein (LDL) cholesterol levels were shared with hepatocyte caQTLs, consistent with the role of hepatocytes in liver enzyme production and cholesterol metabolism. In a parallel analysis of eQTLs, we identified 10 eQTL signals shared with GWAS signals for liver enzymes, blood cholesterol, or triglyceride levels (r^2^ ≥ 0.5, all of which were detected at r^2^ ≥ 0.8; Table S12). These shared signals identify the chromatin accessible regions and genes that may be involved in the genetic regulation of cardiometabolic diseases.

**Table 2:** GWAS signals shared with hepatocyte caQTLs

**Trait**	**Total GWAS signals analyzed**	**GWAS signals shared with caQTLs (%)**
Gamma-glutamyl transferase^3^	200	26 (13%)
Alanine transaminase^3^	149	16 (11%)
Alkaline phosphatase^3^	288	27 (9%)
2-h glucose^4^	12	1 (8%)
Low-density lipoprotein^1^	687	53 (8%)
Total cholesterol^1^	828	59 (7%)
C-reactive protein^63^	438	27 (6%)
Triglycerides^1^	705	37 (5%)
Vitamin D^62^	142	7 (5%)
Body mass index^61^	940	40 (4%)
High-density lipoprotein^1^	814	35 (4%)
Hemoglobin A1c^4^	96	3 (3%)
Type 2 diabetes^5^	402	12 (3%)
Coronary artery disease^2^	241	6 (2%)
Fasting glucose^4^	96	2 (2%)
Waist-to-hip ratio adjusted for BMI^6^	463	11 (2%)

Shared signals are the number of GWAS signals for which the lead variant had moderate LD (r^2^ ≥ 0.5) with the lead variant of a hepatocyte caQTL.

**Figure 4:**
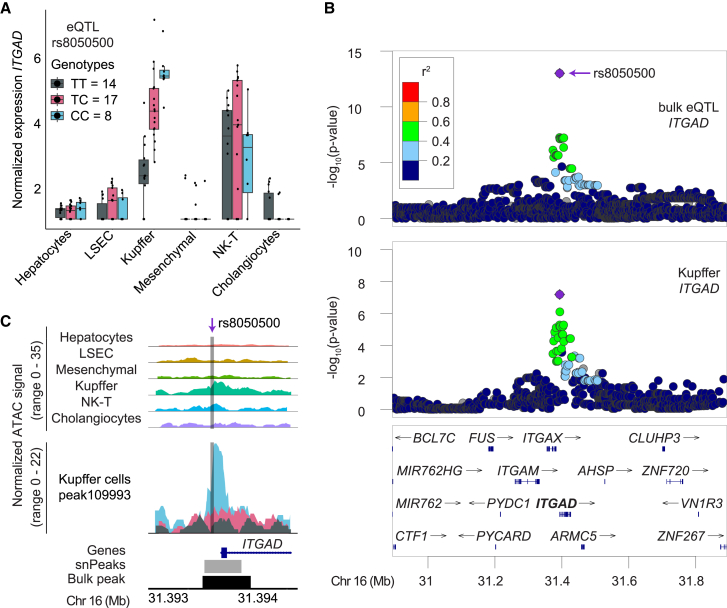
Cell-type eQTL signal shared with a GWAS signal for platelet and reticulocyte counts (A) rs8050500 is associated with *ITGAD* expression only in Kupffer cells. Boxplots show normalized expression levels by genotypes, with individual points representing samples. (B) Shared eQTL signal for *ITGAD* detected in bulk liver (GTEx) and in Kupffer cells. The lead variant rs8050500 (purple dot) in both signals is also associated with platelet^74^ and reticulocyte^75^ counts (data not shown). (C) Coverage plot showing normalized ATAC-seq signal across cell types (top) and in an expanded view of Kupffer cells stratified by genotype rs8050500 (bottom). At the *ITGAD* promoter, individuals with the rs8050500 CC genotype showed more accessible chromatin in Kupffer cells than the TC or TT genotypes. The colors correspond to the genotypes shown in (A).

To identify cell-type eQTL and caQTL signals shared with a broader set of GWAS traits, we evaluated signals from the GWAS Catalog.^64^ We found 394 cell-type caQTLs shared with GWAS signals for 2,127 traits (r^2^ ≥ 0.5, 232 caQTLs for 1,558 traits at r^2^ ≥ 0.8). Similarly, we found 26 cell-type eQTLs shared with ∼196 traits (r^2^ ≥ 0.5, 20 eQTLs for ∼149 traits at r^2^ ≥ 0.8; Table S13). For example, we identified an eQTL in Kupffer cells for *ITGAD* that shared its lead variant with a GWAS signal for platelet^74^ and reticulocyte^75^ counts (Figure 4). *ITGAD* encodes an alpha integrin that forms part of the integrin receptors expressed on Kupffer cells.^76^ The eQTL lead variant resided in the *ITGAD* promoter region, which was most accessible in Kupffer cells. The rs8050500 C-allele was associated with increased *ITGAD* expression and higher platelet and reticulocyte counts. This signal was also present in bulk liver eQTLs for *ITGAD*. This example and others (Table S13) show that cell-type eQTLs and caQTLs for less abundant cell types can inform mechanisms for a wide range of complex traits.

**Figure 5:**
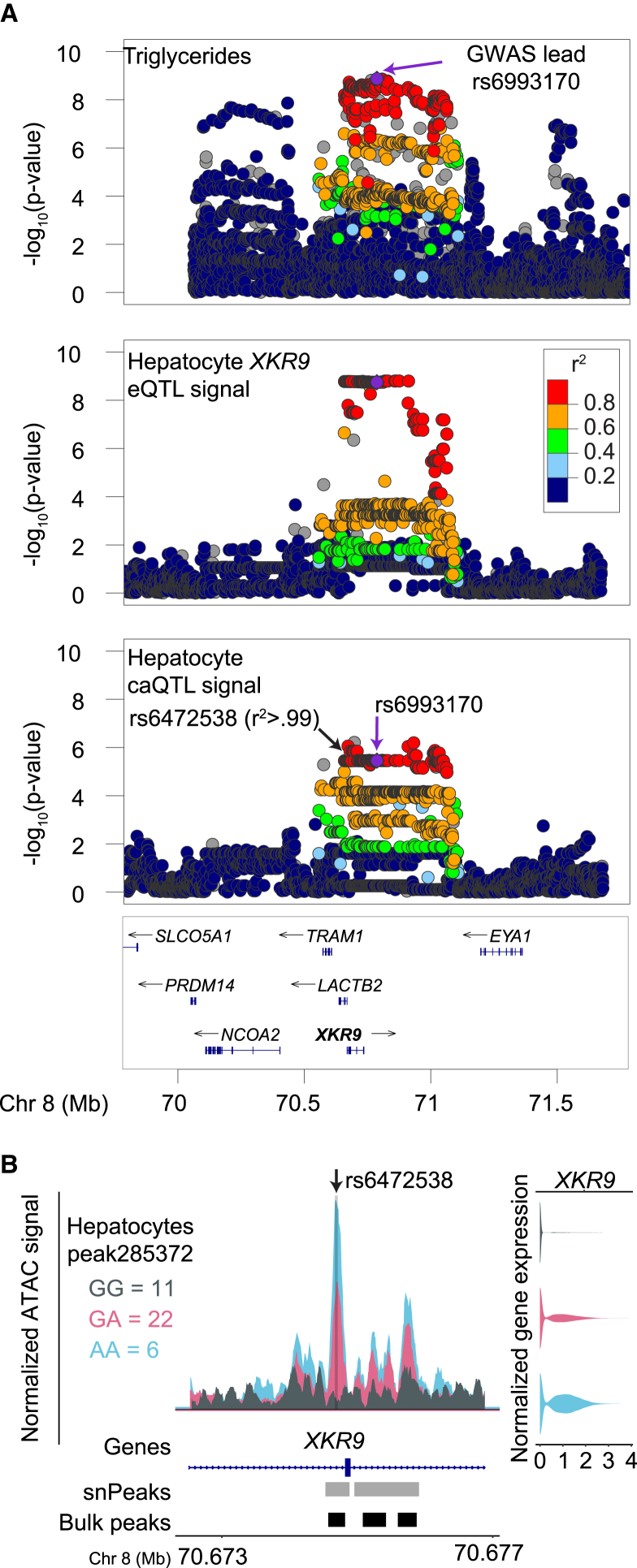
Cell-type caQTL signal shared with a cell-type eQTL signal and a GWAS signal for triglycerides (A) A GWAS signal for triglycerides (lead variant rs6993170) is shared with a hepatocyte eQTL for *XKR9* and a hepatocyte caQTL. One LD proxy variant for rs6993170 is rs6472538 (r^2^ > 0.99). (B) Coverage plot showing normalized ATAC-seq signal in hepatocytes stratified by genotype of rs6472538, located in an *XKR9* intron. Individuals with the rs6472538 AA genotype showed more hepatocyte accessible chromatin and higher hepatocyte gene expression than individuals with the GA or GG genotypes. The gray bars on the bottom indicate regions defined as peaks in hepatocytes, and black bars indicate regions defined as peaks in bulk liver tissue.

GWAS signals shared with both cell-type eQTLs and caQTLs reveal regulatory elements potentially regulating the expression of genes involved in cardiometabolic traits. At the *XKR9* locus, we identified a hepatocyte caQTL shared with a hepatocyte eQTL for *XKR9* (r^2^ = 0.97) and a GWAS signal for triglycerides (r^2^ = 0.87; Figure 5; Table S14); these signals were also observed in bulk liver QTL studies^10^^,^^11^ (Figure S12). The caQTL proxy variant rs6472538 (r^2^ = 0.99) resides within 30 bp of the caPeak center, and the rs6472538 A-allele was associated with increased chromatin accessibility, increased *XKR9* expression, and elevated triglyceride levels. XKR9 functions as a phospholipid scramblase that promotes phosphatidylserine exposure on the surface of apoptotic cells.^77^ These shared signals provide a putative functional variant, altered regulatory element, target gene, and cell type of action for this triglyceride’s GWAS signal. We additionally found 80 hepatocyte caQTLs that were shared with bulk liver eQTLs for 87 genes and a GWAS signal for cardiometabolic traits (r^2^ ≥ 0.5, 67 caQTLs for 65 genes at r^2^ ≥ 0.8; Table S9). This integrative approach demonstrates that combining cell-type chromatin accessibility signals with cell-type and bulk gene expression signals can help fine-map potential functional variants, regulatory regions, and disease-associated genes.

### Cell-type annotation of bulk liver accessible chromatin

**Figure 6:**
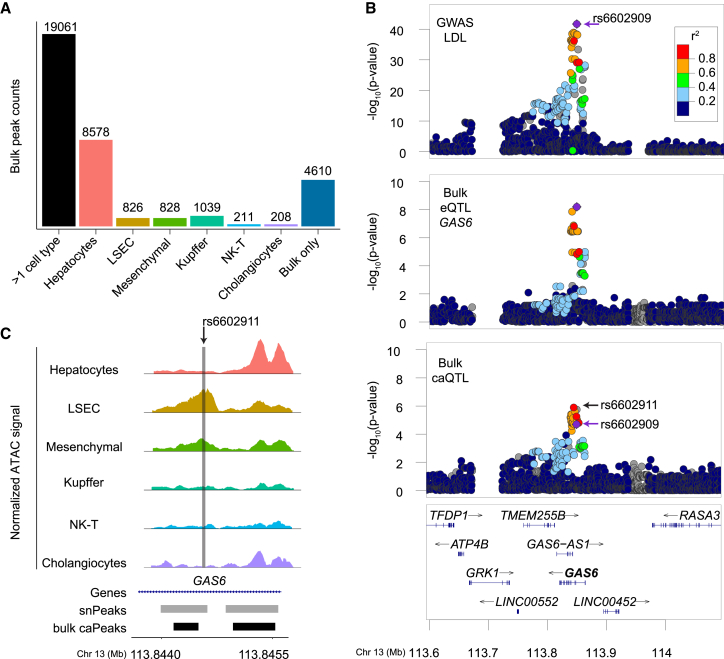
Liver cell types that correspond to bulk caQTLs and a subset that is colocalized with one or more GWAS signals (A) Distribution of bulk caPeaks based on the cell types in which the same region of chromatin was accessible. Most peaks were detected in multiple cell types (19,061) or only in hepatocytes (8,578). 3,112 caQTLs were only detected in less abundant cell types. A subset (917) of these 30,751 bulk caPeaks had caQTLs that colocalized with cardiometabolic GWAS signals. (B) GWAS signal for LDL with lead variant rs6602909 is colocalized with a bulk eQTL for *GAS6* and a bulk caQTL conditioned on rs74118417 (material and methods). One LD proxy variant for rs6602909 is rs6602911 (r^2^ = 0.82). (C) The bulk caQTL lead variant rs6602911 overlaps accessible chromatin in LSECs and mesenchymal cells. The coverage plot shows a normalized ATAC-seq signal at the *GAS6* locus for each cell type.

A key advantage of cell-type resolution of chromatin profiles is the ability to annotate cell types for caQTLs detected in bulk tissue analyses. Using cell-type data, we were able to annotate cell types for 87% (30,751) of the bulk peaks^11^ (Data S2). While the majority of bulk caPeaks (62%) were detected across multiple cell types, we detected 3,112 bulk caPeaks in only a single non-hepatocyte cell type (Figure 6; Table S15). This finding underscores the value of cell-type annotation for bulk caPeaks, enabling regulatory signals to be predicted in less abundant cell types even without identifying cell-type QTLs.

Within complex tissues such as the liver, each cell type contributes differently to physiological homeostasis and disease pathogenesis. Mapping GWAS loci to regulatory elements of these functionally distinct cell types helps uncover relevant mechanisms for liver diseases. Thus, we annotated the 998 bulk caQTLs^11^ that colocalized with GWAS signals of cardiometabolic traits by overlapping these bulk caPeaks with cell-type peaks. We identified putative cell type(s) for 917 of these bulk caPeaks, and the distribution of these colocalized bulk caQTLs across different cell types corresponded with cell-type proportions in our dataset (Table S16). One example is for a bulk caQTL for a peak detected only in LSECs and mesenchymal cells and that colocalized with GWAS signals for alkaline phosphatase (ALP), total and LDL cholesterol, and triglyceride levels (Figure 6). The bulk caPeak lies in the intron of *GAS6*, and its lead variant also colocalized with a bulk liver eQTL for the same gene. *GAS6* has been implicated in hepatic stellate cell activation,^78^ consistent with the role of mesenchymal cells in fibrosis. Overall, these analyses highlight the importance of cell-type resolution in nominating cell types for signals identified in bulk analyses, particularly for less abundant cell populations.

## Discussion

We profiled the human liver at single-nucleus resolution and revealed cell-type regulatory landscapes relevant to liver-related and cardiometabolic traits. Through multiome analysis of snRNA-seq and snATAC-seq data from 39 human liver samples, we identified distinct regulatory profiles across six major cell types, detecting 1,885 caQTLs and 67 eQTLs. This cell-type resolution improves our understanding of liver genetics by suggesting the cellular context in which disease-associated variants operate. We showed that a single-nucleus approach can help dissect how genetic variants contribute to various physiological and pathological conditions, such as metabolic disorders in hepatocytes and inflammatory responses in Kupffer cells. By linking cell-type regulatory elements to GWAS signals and annotating previously identified bulk tissue associations, we can predict regulatory mechanisms underlying disease associations that are obscured in conventional bulk tissue analyses.

Our cell-type resolved QTL analyses suggested the cellular origins of regulatory mechanisms underlying several GWAS associations for liver-related traits. We identified an eQTL for *ITGAD* in Kupffer cells, which showed that genetic variation may influence integrin-mediated leukocyte adhesion in liver-resident macrophages. Additionally, integrins participate in the activation and expansion of Kupffer cells in non-alcoholic steatohepatitis.^76^ The shared genetic architecture between these cell-type eQTLs and GWAS signals enhances our understanding of GWAS loci by providing biologically interpretable mechanisms to statistical associations, pinpointing the cellular origins of these regulatory effects.

Our cell-type approach demonstrates value for annotating regulatory elements identified in bulk liver studies. Despite a modest size of 39 samples, we successfully detected 70% of the accessible chromatin regions detected in a bulk liver chromatin accessibility study of 138 samples^11^ while identifying 70,884 cell-type peaks undetected in bulk data. Most of these novel peaks were detected only in non-hepatocyte cell types, highlighting that single-nucleus profiling effectively captures regulatory elements masked by cellular heterogeneity in bulk analyses. By annotating cell types for 30,751 previously reported bulk liver caQTLs,^11^ we enhanced the interpretation of regulatory signals, including a subset of 917 regulatory elements at GWAS signals, providing cellular context for disease-relevant genetic variation. For example, we annotated the accessibility of a bulk caPeak to LSECs and mesenchymal cells located within an intron of *GAS6*. This bulk caQTL also colocalized with an eQTL for the same gene, providing mechanistic insight into how genetic variation might influence fibrotic processes in a cell-type manner. *GAS6* encodes growth arrest-specific 6, a signaling hub of liver fibrosis,^79^ and mice deficient in *Gas6* show less susceptibility to steatosis and fibrosis.^78^ *GAS6* is involved in cellular differentiation, the immune response, and the activation of hepatic stellate cells.^78^ The association of this signal with elevated ALP levels and other cardiometabolic traits underscores its importance in metabolic liver diseases and may offer a promising target for anti-fibrotic therapies.

While this study demonstrates the value of cell-type resolved regulatory analysis in liver tissue, several limitations shape opportunities for future research. Due to statistical power constraints, we cannot definitively establish cell-type specificity of QTLs, as the absence of detected signals in less abundant cell populations may reflect insufficient detection sensitivity rather than true biological absence of regulatory effects. Our attempts to identify additional eQTLs by lowering UMI thresholds during nuclei filtration produced fewer significant results, likely due to the inclusion of non-nucleus barcodes or contaminated nuclei (data not shown). Our power analysis suggests that increasing the sample size will yield more eQTLs than adding more nuclei per sample. The benefit of larger cell counts was restricted to less abundant cell types. For these cell types, a dual strategy of increasing donors and using targeted capture of nuclei^80^^,^^81^ may help achieve adequate statistical power. Larger sample size studies would allow for increased detection of signals and more colocalizations with GWAS signals.^82^ Future studies including more individuals with defined disease states would enable better identification of context-dependent regulatory effects. Integrating emerging technologies such as cell-type Hi-C with snATAC-seq and snRNA-seq offers promising approaches for connecting regulatory elements to target genes through direct chromatin contact. Finally, our study offers opportunities for future research to functionally validate the predicted QTL signals in their respective cell types.

Despite these limitations, our findings demonstrate the utility of single-nucleus approaches for characterizing the genetic variation in liver cell types. Coupling single-nucleus and bulk profiling enhanced our ability to dissect the cellular contexts of genetic regulation in the liver and identified cell-type regulatory elements relevant to cardiometabolic disease.

## Data and code availability

The accession number for the raw and processed genotype, snATAC-seq, and snRNA-seq data reported in this paper is GEO: GSE296875.

## Acknowledgments

This study was supported by 10.13039/100000002NIH grants R01DK072193, UM1DK126185, and T32HL069768 (H.J.P.). We thank the donors of liver samples, which were obtained from the LTCDS supported by NIH contract N01DK70004/HHSN267200700004C. We thank members of the Mohlke lab for helpful discussions on computational analyses. We gratefully acknowledge the technical support from University of North Carolina (10.13039/100006808UNC) Cores. The UNC High Throughput Sequencing Facility is supported by the University Cancer Research Fund, a 10.13039/100016516Comprehensive Cancer Center Core Support grant (NIH P30 CA016086), and the UNC Center for Mental Health and Susceptibility grant (NIH P30 ES010126). The Advanced Analytics Core is funded through the UNC Center for Gastrointestinal Biology and Disease grant (NIH P30 DK034987). The UNC Pathology Services Core Facility is supported in part by an NCI Center Core Support Grant (NIH P30 CA016086). We also thank GTEx for liver tissue gene expression data. The GTEx Project was supported by the Common Fund of the Office of the Director of the NIH and by 10.13039/100000054NCI, 10.13039/100000051NHGRI, 10.13039/100000050NHLBI, 10.13039/100000026NIDA, 10.13039/100000025NIMH, and 10.13039/100000065NINDS. The data used for the analyses described in this manuscript were obtained from the GTEx Portal.

## Declaration of interests

F.I. is a patent holder of *UGT1A1* genetic testing, a patent holder of genetic testing for drug-induced proteinuria and hypertension, a stockholder of AbbVie stocks, a stockholder of BeOne stocks, and a BeOne employee.

## Supplemental information

Data S1. Tables S1–S16

Data S2. Characteristics of liver cell-type peaks

Data S3. Pairwise cell-type marker peaks

Document S2. Article plus supplemental information
