# Supplementary material for: Liver single-nucleus multiome profiling reveals cell-type mechanisms for cardiometabolic traits
Source: Am J Hum Genet. 2025 Dec 2;113(1):133–48. doi: 10.1016/j.ajhg.2025.11.009 (PMC12805840; doi:10.1016/j.ajhg.2025.11.009)
Supplement: Document S1. Figures S1–S12 [file mmc1.pdf]

The American Journal of Human Genetics, Volume *113*

Supplemental information

Liver single-nucleus multiome profiling reveals cell-type mechanisms for cardiometabolic traits

Abdalla A. Alkhawaja, Kevin W. Currin, Hannah J. Perrin, Swarooparani Vadlamudi, Amy S. Etheridge, K. Alaine Broadaway, Gabrielle H. Cannon, Carlton W. Anderson, Anne H. Moxley, Alina C. Iuga, Erin G. Schuetz, Federico Innocenti, Terrence S. Furey, and Karen L. Mohlke

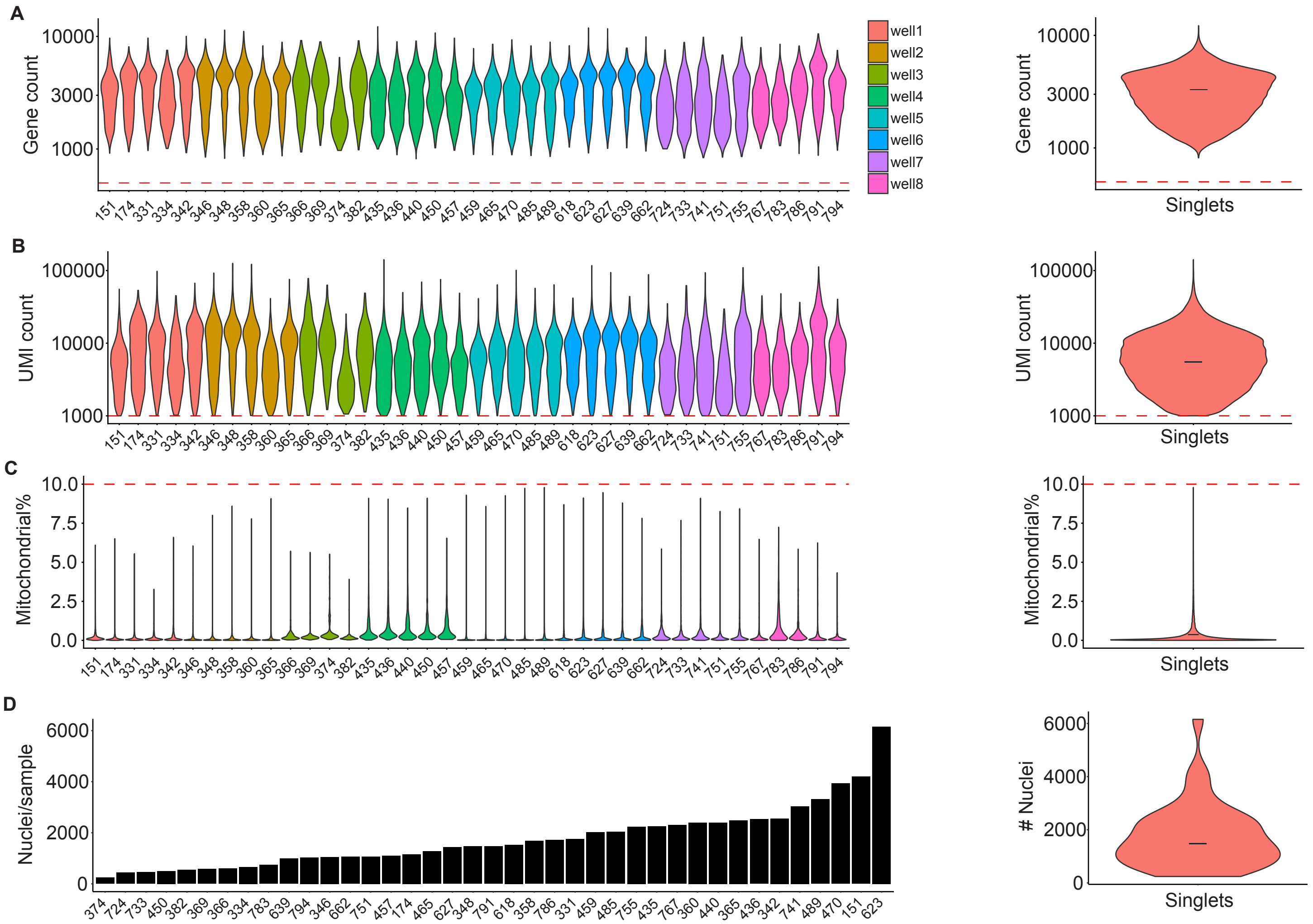

Figure S1: Quality of single-nucleus RNA sequencing data. Data for each metric is shown by sample, colored by processing batch (wells 1-8, left panels), and aggregated across all nuclei (right panels). (A) Gene count distribution per nucleus. (B) Unique molecular identifier (UMI) count distribution per nucleus. (C) Mitochondrial RNA content as percentage of total counts per nucleus. (D) Post-quality control nucleus count per donor sample and distribution across donors. n = 39 samples. Quality control thresholds are indicated by red dashed lines.

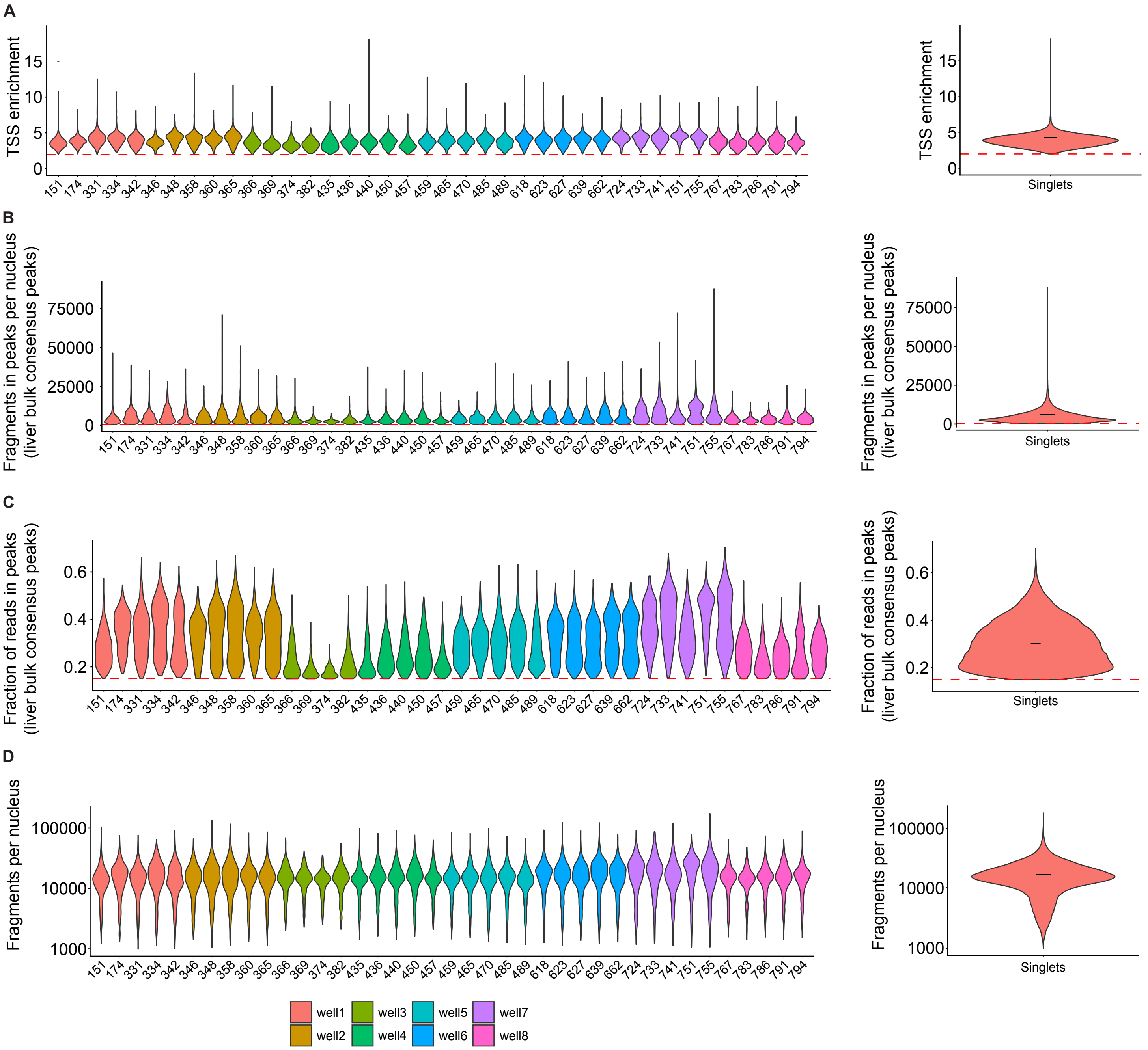

Figure S2: Quality of single-nucleus ATAC sequencing data. Data for each metric is shown by sample, colored by processing batch (wells 1-8, left panels), aggregated across all nuclei (right panels). (A) Transcription start site (TSS) enrichment scores per nucleus calculated using EnsDb.Hsapiens.v86. (B) Number of fragments in peaks per nucleus. (C) Fraction of fragments in peaks (FRiP) per nucleus. Thresholds in panels (B and C) were applied to peaks with coordinates from bulk liver tissue. (D) Total fragment count distribution per nucleus. n = 39 samples. Quality control thresholds are indicated by red dashed lines.

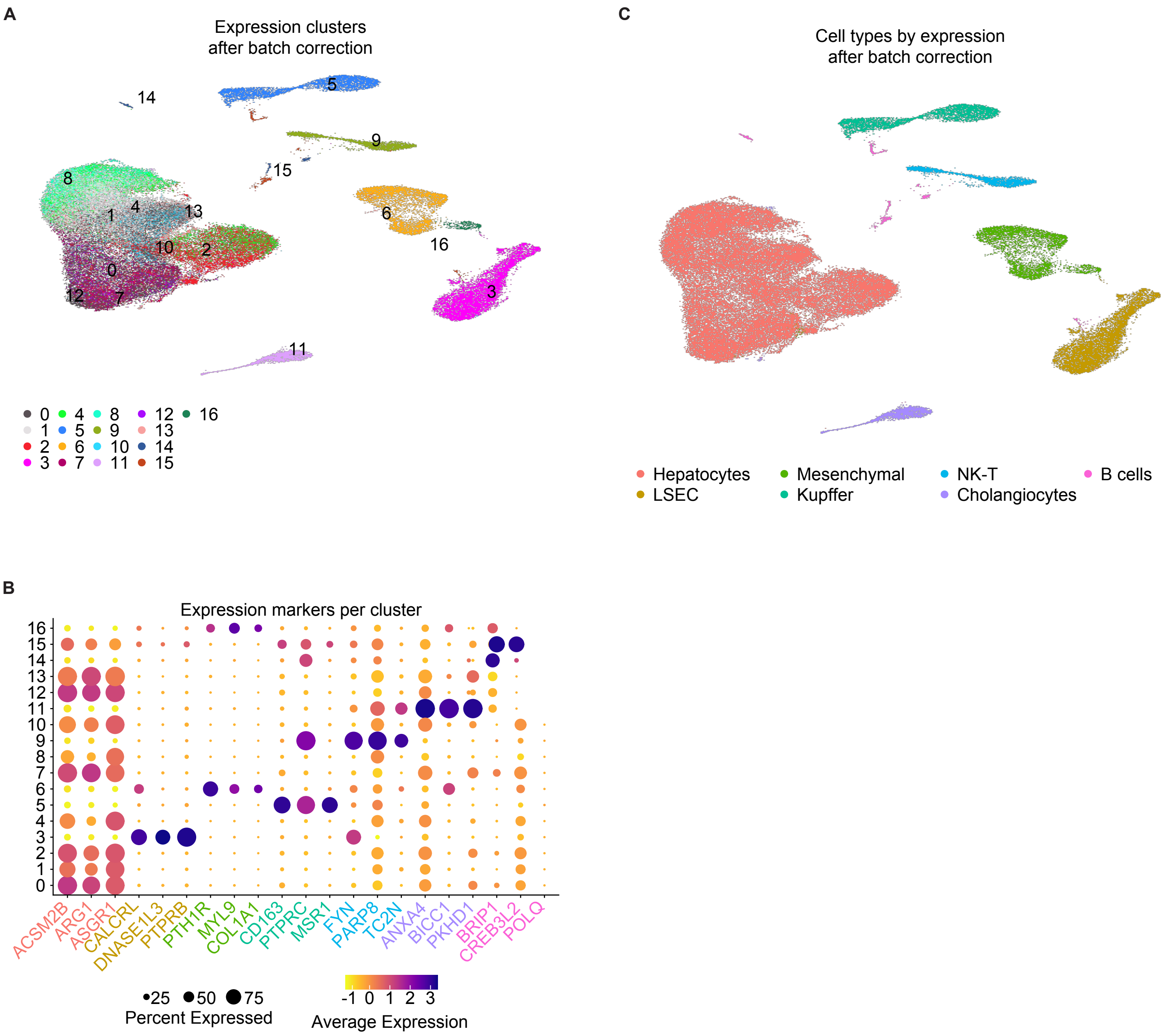

Figure S3: Cell type identification using single-nucleus RNA-seq data. (A) Initial clustering of expression data after batch correction showing 17 distinct clusters. (B) Established cell-type marker gene expression across identified clusters. Dot size represents the percentage of cells expressing the marker and color intensity shows the average expression level. (C) Cell-type annotations after batch effect correction of snRNA-seq data.

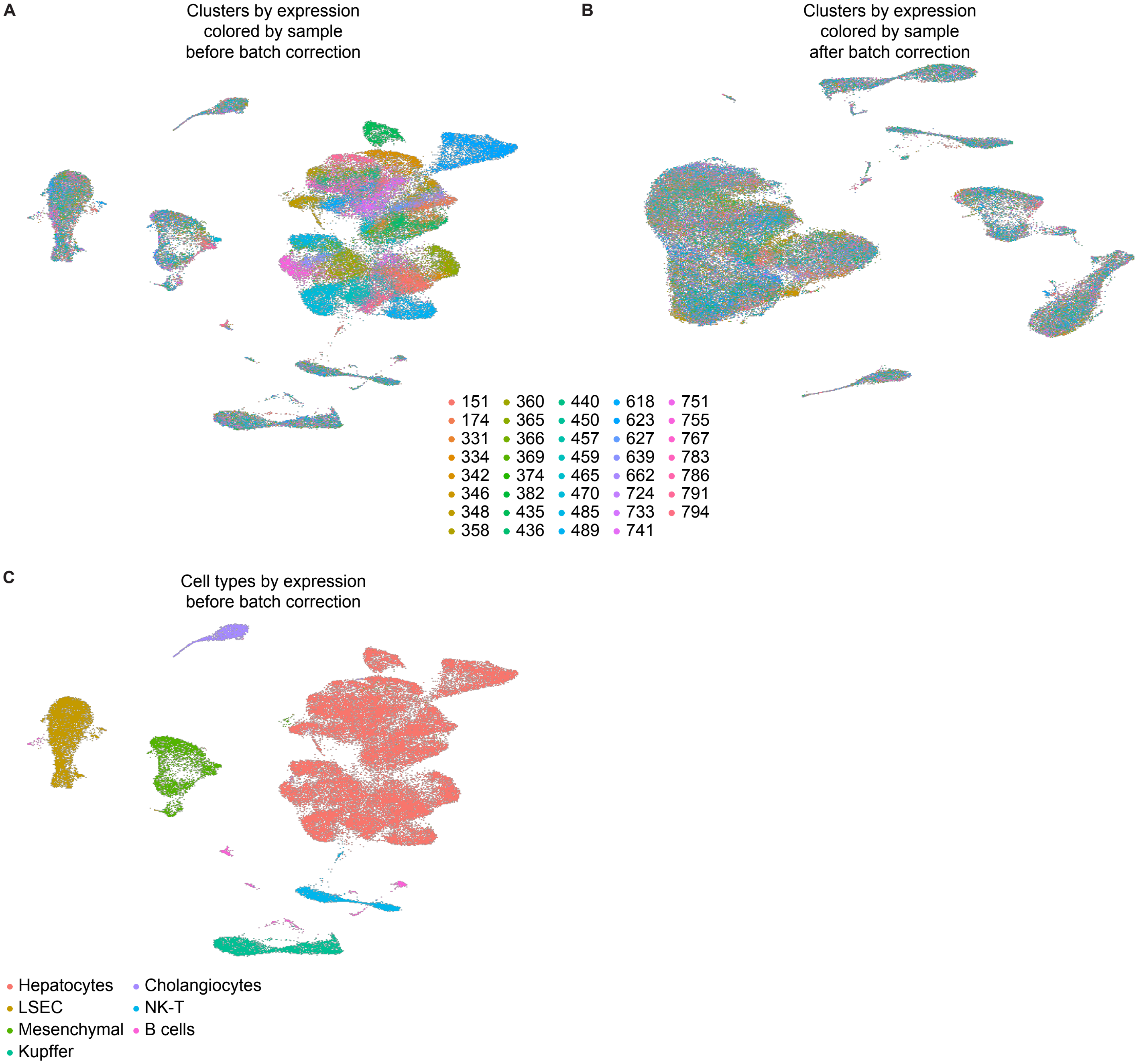

Figure S4: Batch correction in single-nucleus RNA-seq data. (A - B) Clusters of expression data before (A) and after (B) batch correction colored by sample. (C) Cell-type annotations prior to batch correction of snRNA-seq data. Cell-type annotation after batch correction of snRNA-seq data is shown in Figure S3.

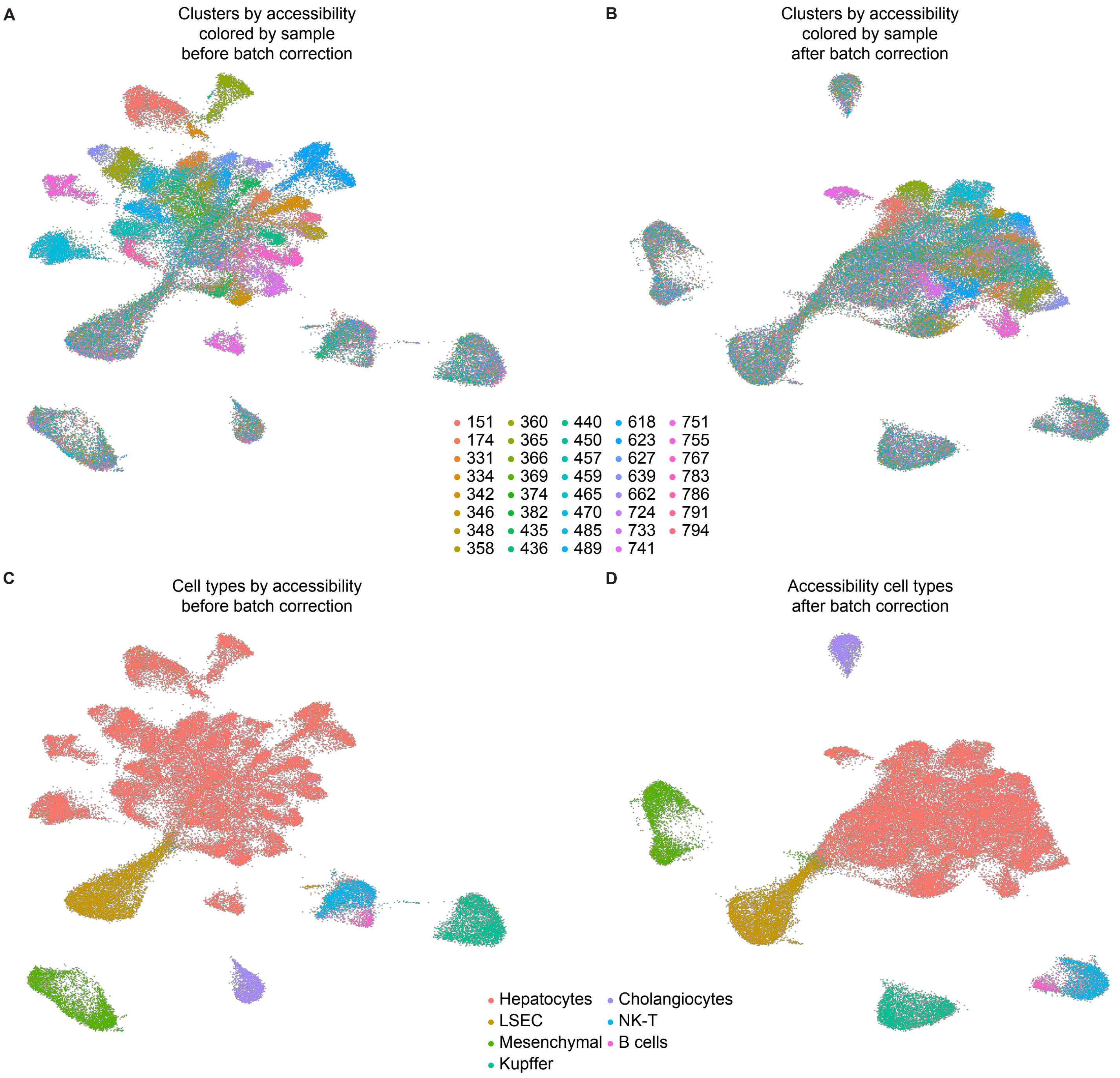

Figure S5: Batch correction in single-nucleus ATAC-seq data. (A - B) Clusters of expression data before (A) and after (B) batch correction colored by sample. (C - D) Cell-type annotations before (C) and after (D) batch correction of snRNA-seq data. 20 harmonized LSI components were used to recalculate clusters and UMAP projections.

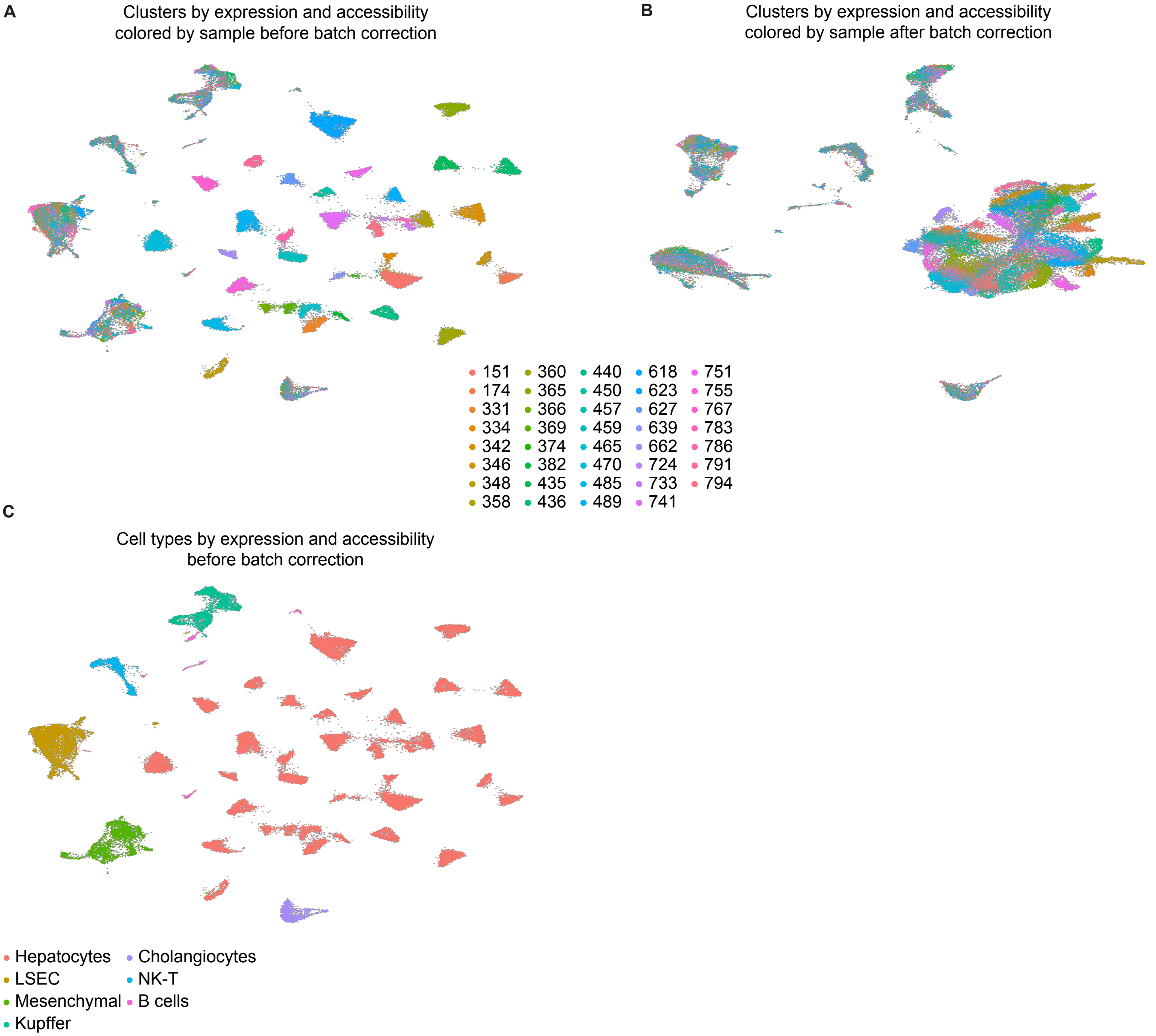

Figure S6: Batch correction in joint UMAP of single-nucleus RNA and ATAC data. (A - B) Clusters of joint profiles (snRNA-seq + snATAC-seq) before (A) and after (B) batch correction colored by sample. (C) Cell-type annotations of joint profiles before batch correction. Cell-type annotations of joint profiles after batch correction is shown in Figure 1.

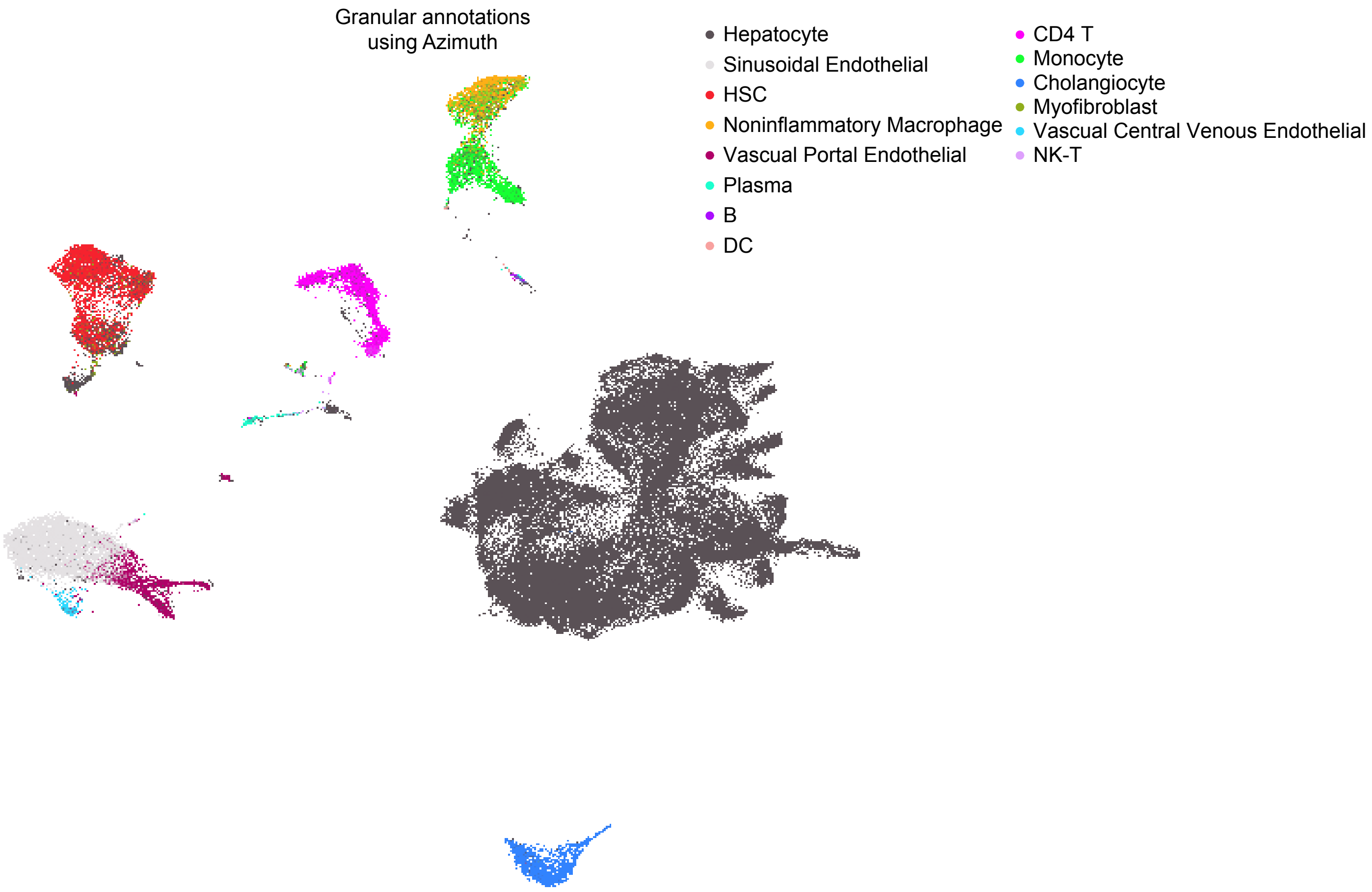

Figure S7: Granular cell-type annotations. Detailed annotation of batch-corrected and snRNA+snATAC-seq integrated data using Azimuth reference mapping.

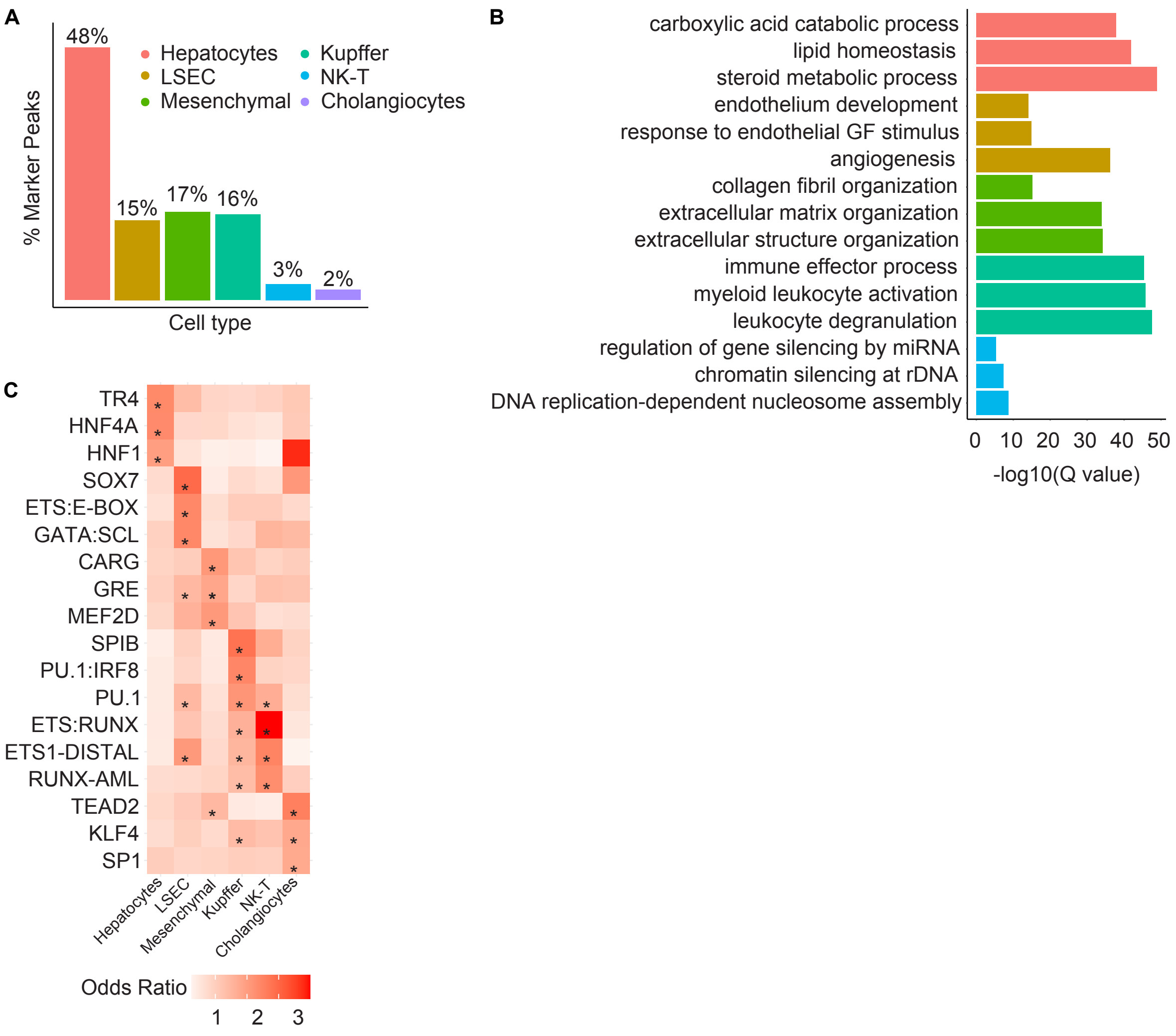

Figure S8: Cell-type marker peaks and their associated regulatory features. (A) Distribution of cell type marker peaks across major liver cell populations, ordered by cell-type proportion. Among 17,147 marker peaks, most are hepatocytes (48%), followed by mesenchymal cells (17%), Kupffer cells (16%), and LSECs (15%), while NK-T cells (3%) and cholangiocytes (2%) show fewer marker peaks. (B) Gene ontology terms of genes near marker peaks based on GREAT. The x-axis represents statistical significance as -log10(Q-value), and the three most significant terms per cell type are displayed. Bar colors denote cell types as in (A). (C) Heatmap showing transcription factor (TF) motif enrichment within marker peaks for each cell type, quantified by enrichment odds ratios (color intensity). Asterisks denote significant enrichment (FDR < 0.05). The three most enriched motifs per cell type are shown.

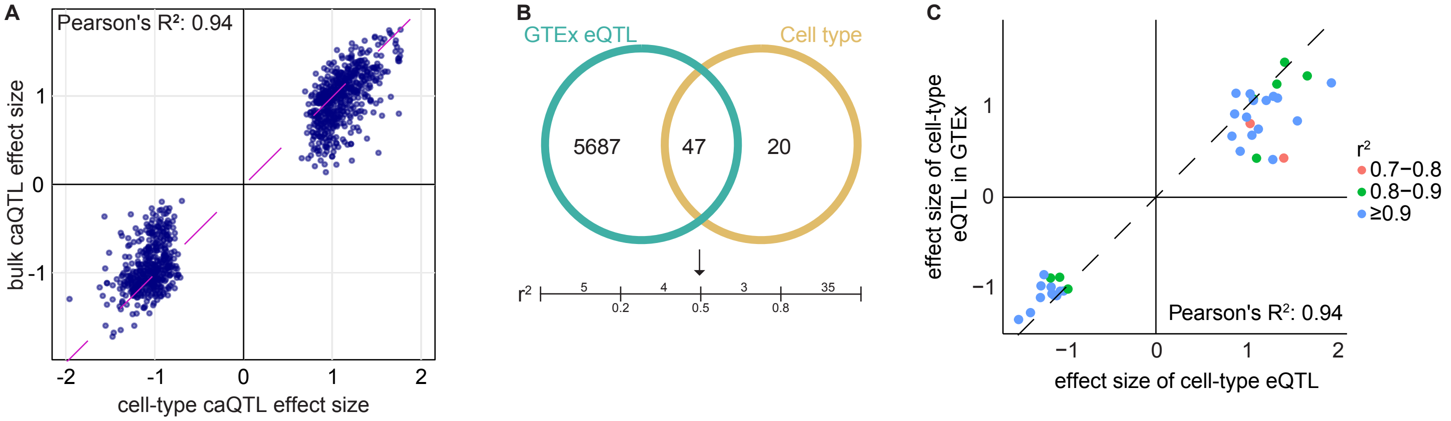

Figure S9: Comparison between bulk and cell-type QTLs. (A) The effect sizes (beta) of caQTLs identified in both the bulk liver tissue and one or more cell-type show strong concordance (Pearson's $R^2 = 0.94$). Dots represents the 975 caQTLs that are lead variants both in bulk and cell-type data. (B) Venn diagram detailing the LD r^2 between 47 genes with detected eQTLs both in GTEx bulk liver tissue (green) and our cell-type analysis (gold). We considered eQTLs with LD $r^2 > .5$ as shared. While most eQTLs were only detected in the GTEx bulk analysis (5,687 genes), we identified 20 novel cell-type eQTLs for 20 genes. Among the 47 eQTLs detected in both datasets, the line plot shows the LD r^2 distribution between lead variants; 38 show strong linkage disequilibrium ($r^2 \geq 0.8$). (C) The effect sizes (beta) of cell-type eQTL lead variants in the single-nucleus study and their effect sizes in GTEx show strong concordance (Pearson's $R^2 = 0.94$). Dots represent 36 cell-type lead variants that were also tested in bulk liver GTEx.

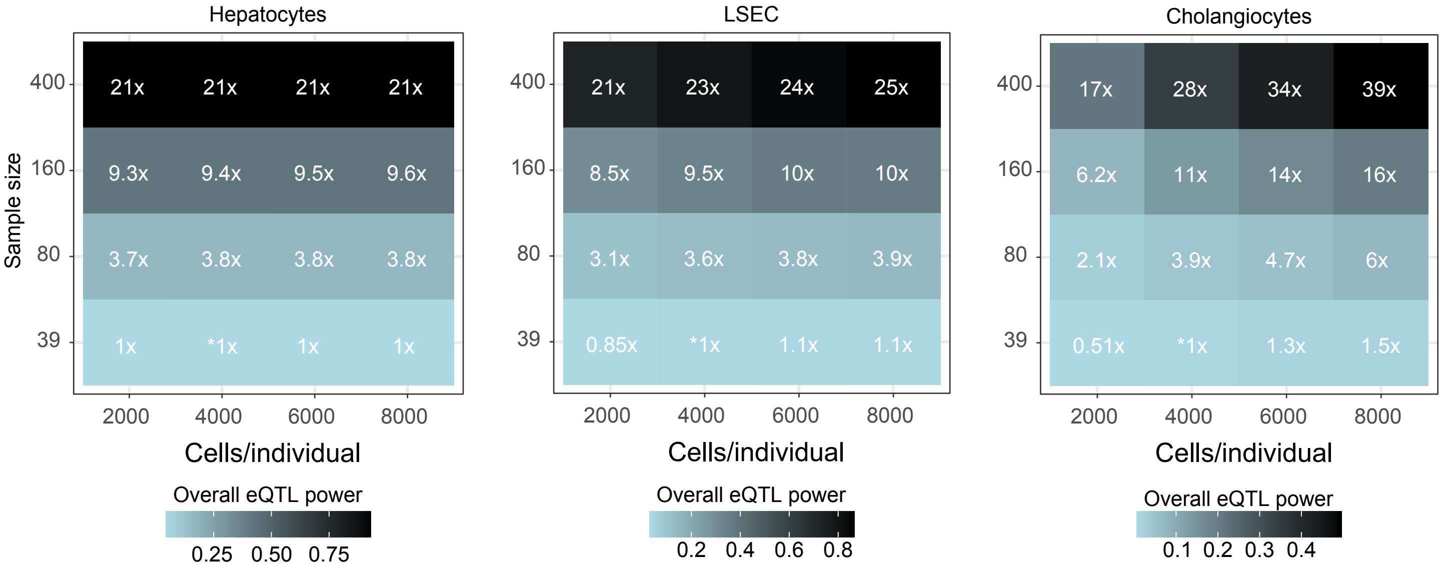

Figure S10: Cell-type eQTL power analysis. Estimated change in eQTLs relative to current study design for high-(hepatocytes), mid-(LSEC) and low-abundance (cholangiocytes) cell types using scPower. Asterisks indicate current study design. Color intensity indicates higher overall eQTL power.

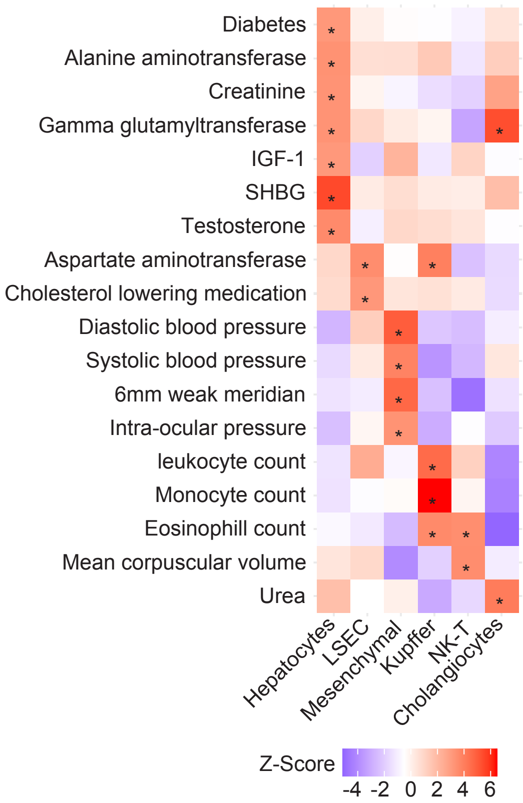

Figure S11: GWAS variants are enriched in cell-type peaks. Enrichment of GWAS signals in cell-type accessible chromatin regions. The heatmap shows z-scores of heritability enrichment across cell types. Asterisks indicate significant enrichments (FDR < 0.05).

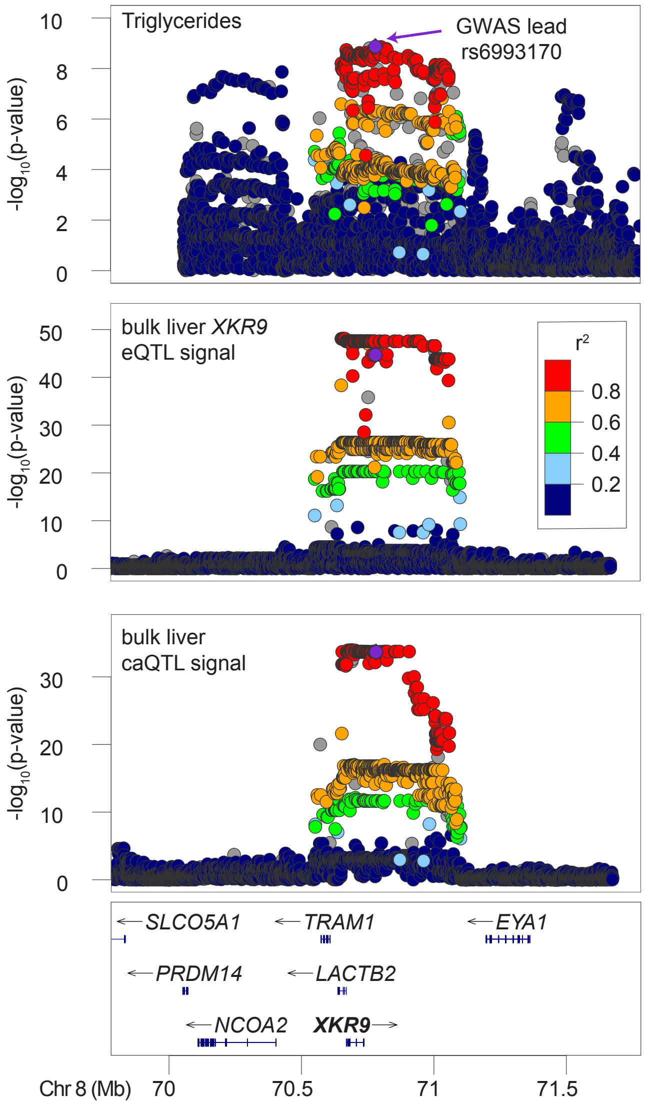

Figure S12: *XKR9* locus genetic variants identified in bulk datasets. A GWAS signal for triglycerides (lead variant rs6993170) is shared with a bulk liver eQTL for *XKR9* (rs13255886, r^2=.99) and a bulk liver caQTL (rs12675271, r^2=.99). The GWAS lead variant is shown by a purple dot in all the plots. The hepatocyte caQTL signal and the eQTL signal for *XKR9* are shown in Figure 5.
